# Hypoxia tolerance, longevity and cancer-resistance in the mole rat *Spalax* – a liver transcriptomics approach

**DOI:** 10.1038/s41598-017-13905-z

**Published:** 2017-10-30

**Authors:** Hanno Schmidt, Assaf Malik, Anne Bicker, Gesa Poetzsch, Aaron Avivi, Imad Shams, Thomas Hankeln

**Affiliations:** 1 Molecular Genetics and Genome Analysis, Institute of Organismic and Molecular Evolution, Johannes Gutenberg-University, Johann Joachim Becher-Weg 30 A, D-55128 Mainz, Germany; 2 Genomic Evolution and Climate, Senckenberg Biodiversity and Climate Research Centre (SBiK-F), D-60325 Frankfurt am Main, Germany; 3 Institute of Evolution, University of Haifa, Mount Carmel, Haifa, 31905 Israel

## Abstract

The blind subterranean mole rat *Spalax* shows a remarkable tolerance to hypoxia, cancer-resistance and longevity. Unravelling the genomic basis of these adaptations will be important for biomedical applications. RNA-Seq gene expression data were obtained from normoxic and hypoxic *Spalax* and rat liver tissue. Hypoxic *Spalax* broadly downregulates genes from major liver function pathways. This energy-saving response is likely a crucial adaptation to low oxygen levels. In contrast, the hypoxia-sensitive rat shows massive upregulation of energy metabolism genes. Candidate genes with plausible connections to the mole rat’s phenotype, such as important key genes related to hypoxia-tolerance, DNA damage repair, tumourigenesis and ageing, are substantially higher expressed in *Spalax* than in rat. Comparative liver transcriptomics highlights the importance of molecular adaptations at the gene regulatory level in *Spalax* and pinpoints a variety of starting points for subsequent functional studies.

## Introduction

Much of our knowledge on biological processes and principles, especially in biomedical research, is based on studies in a rather small number of model organisms such as mouse and rat. However, the applicability of standard animal models e.g. for cancer research is limited^1,2^ and there is a need for a more diverse set of “non-model” organisms showing unusual phenotypes. Two rodents that have attracted attention as emerging models for biomedical research are the blind mole rat *Spalax* and the naked mole rat *Heterocephalus glaber* ^3^; each of which shares a set of unique biological traits highly relevant for human health. Both taxa are tolerant to extreme environmental conditions such as strong hypoxia and reoxygenation. Studying their adaptive mechanisms to prevent hypoxic ischemia or respiratory diseases will be highly interesting. Furthermore, both taxa have extraordinarily long life spans and show strong cancer resistance, making them attractive for ageing and cancer research^4–9^. The less known *Spalax*, however, has an additional advantage: *Spalax* belongs to the same rodent superfamily (Muroidea) as mouse and rat, while *Heterocephalus* has been phylogenetically separated from this clade for more than 70 million years^7,10^. Thus *Spalax* allows for a better comparison of biological traits to established rodent models.

The blind mole rats from the *Spalax* superspecies complex^11,12^ inhabit self-dug burrows in plains from northern Africa over the Middle East to southwestern Asia. The hypoxia tolerance, which the species complex has evolved during millions of years of subterranean life^13^, enables them to tolerate oxygen levels as low as 3% for up to 14 hours without noticeable detrimental effects, while rats survive such hypoxic stress for only 2–3 hours^14^. In natural habitats, oxygen levels of 7.2% were measured in *Spalax* burrows after heavy rainfalls^15^, confirming the ecological relevance of effective adaptation to strong environmental hypoxia. While the life span of rats is approximately four years, *Spalax* can reach ages of 20 years and more^16^. At present *Spalax* is the only species where no spontaneous tumour formation has been detected in thousands of individuals studied in the past 40 years. With only a few exceptions, carcinogenic substances were not able to induce malignancies, while all similarly treated mice and rats developed tumours^6^. Moreover, fibroblasts of *Spalax* secrete substance/s, yet to be identified, which lead to the death of cancer cells from different species, including a wide range of human cancer cells^6^. Interferon-β secretion has been speculated to be involved in *Spalax* cancer resistance^17^, but these results have been criticized^6^. While in *Heterocephalus* the longevity and linked cancer resistance have been ascribed at least partially to the species’ eusociality^18^, these traits in the solitary living *Spalax* have probably evolved by convergence using different molecular mechanisms. Genome sequencing of *Spalax* recently unravelled interesting genomic features, which potentially form the basis of some of the observed adaptive traits^7^. Observations included high rates of DNA editing, reduced chromosomal rearrangements, adaptation to darkness by pseudogenisation of visual system genes, adaptation to hypoxic/hypercapnic conditions by positive selection in respiratory proteins and reduced sensitivity to hypercapnia-induced acid pain^7^. A hypoxia-induced upregulation of SINE B1 and LINE-1 retrotransposon expression was noticed, which could possibly play a role in the cancer resistance of *Spalax*: In the presence of a naturally occurring substitution in the *TP53* tumour suppressor gene^19^, the TRAIN (‘transcription of repeats activates interferon’) system is activated, resulting in diminished apoptosis but increased necrosis in *Spalax*, as well as an increase in cell cycle arrest/DNA repair^7,19^. Further insight into the molecular basis of *Spalax*’ adaptations has been gained on the transcriptome level by microarray studies of brain^16^ and muscle tissues^16,20^, inferring differentially expressed genes at hypoxia and normoxia. In these experiments, biological processes and pathways altered by hypoxia in the gene expression of *Spalax* included angiogenesis, apoptosis, cancer, embryonic/sexual development, epidermal growth factor receptor binding, coordinated suppression and activation of distinct groups of transcription factors, membrane receptors and oxidative stress management. In a recent RNA-Seq study on brain tissue, normoxic mRNA levels of genes associated with DNA damage repair were expressed significantly higher in *Spalax* when compared to rat, while genes associated with bioenergetics were expressed at lower levels^21^.

To achieve a more comprehensive understanding of *Spalax*’ stress adaptation phenotype, it is mandatory to study regulated genes and pathways active in additional organs of the body. The liver is indispensable to life in all vertebrates, and its response to hypoxia is crucial for the organism, as the organ exerts highly oxygen-dependent functions such as glycolysis/gluconeogenesis and the detoxification of harmful endogenous or xenobiotic substances^22^. Accordingly, hypoxia can cause severe pathological phenotypes in the liver of non-adapted organisms, including inflammation and carcinogenesis^23^. Comparing *Heterocephalus* liver transcriptomes to those of mouse, an increased expression of DNA repair signalling genes has recently been observed^4^. In *Spalax* however, our knowledge of differential gene expression in liver tissue is very limited so far. Respiratory proteins and proteins of the anti-oxidant defence indeed suggested the existence of species-specific, possibly adaptive gene regulatory expression patterns in *Spalax* liver^24,25^, confirming the need for a transcriptome-wide analysis. The gene expression profiles of normoxic and hypoxic livers from *Spalax* and rat specimens were therefore investigated by Illumina-based mRNA-Seq. The aim of the present study was to reveal *Spalax*-specific gene-regulatory strategies (as opposed to rat) to cope with strong, acute hypoxia in liver tissue. In addition we aimed to identify genes which are differentially expressed constitutively between *Spalax* and rat liver tissue under normoxic, non-stress conditions. These data may help unravel the genetic basis of traits associated with cancer-resistance and longevity in *Spalax*.

## Results

### Sequencing and raw data processing

In order to infer mRNA gene expression, we performed Illumina-based deep-sequencing of transcriptomes, producing around 257 million sequence reads for *Spalax* and 327 million reads for rat. In both species, on average 93% of the reads passed quality processing, resulting in a total of 240 million cleaned *Spalax* reads and 303 million cleaned reads from rat. On average, 97% of the 121 million *Heterocephalus* reads passed the same quality processing pipeline, resulting in 117 million cleaned reads for *Heterocephalus* and 660 million cleaned reads overall (Additional Table 1).

**Table 1:** GO term enrichment in response to hypoxia.

	***Spalax***		**rat**	
	**upregulated genes**	**downregulated genes**	**upregulated genes**	**downregulated genes**
# overrepresented GO terms	75	0	84	6
# underrepresented GO terms	9	5	13	14

Shown are the numbers of significantly over- and underrepresented GO terms in the groups of up- and downregulated genes after hypoxia exposure in *Spalax* and rat.

### Read-mapping and differential gene expression analysis

Expression levels of individual genes were obtained by bioinformatical mapping of the cDNA-derived sequence reads to annotated reference genomes, followed by read-counting. Normalized gene expression was expressed as FPKM values ( = fragments per kilobase of exon sequence per million of mapped fragments). Thereby, we could detect expression in at least one of the replicates for 14,380 genes in *Spalax* and 15,033 genes in rat (Supplementary dataset 1). Correlation of the FPKM values between the two species under normoxia was moderate with a coefficient of determination of 0.69. Under normoxia, 9,173 genes were expressed in all three rodent species (Additional Fig. 1). Among the top 20 most highly expressed genes under normoxic conditions, twelve were found in *Spalax* and rat, with the serum transporter transthyretin (*TTR*) being the highest expressed gene in both species (Supplementary dataset 2). Noteworthy, n = 1227 genes showed at least a 10-fold higher expression in *Spalax* than in rat, while only 461 genes were >10-fold higher expressed in rat compared to *Spalax*. This quantitative relation did not change under hypoxia.

**Figure 1:**
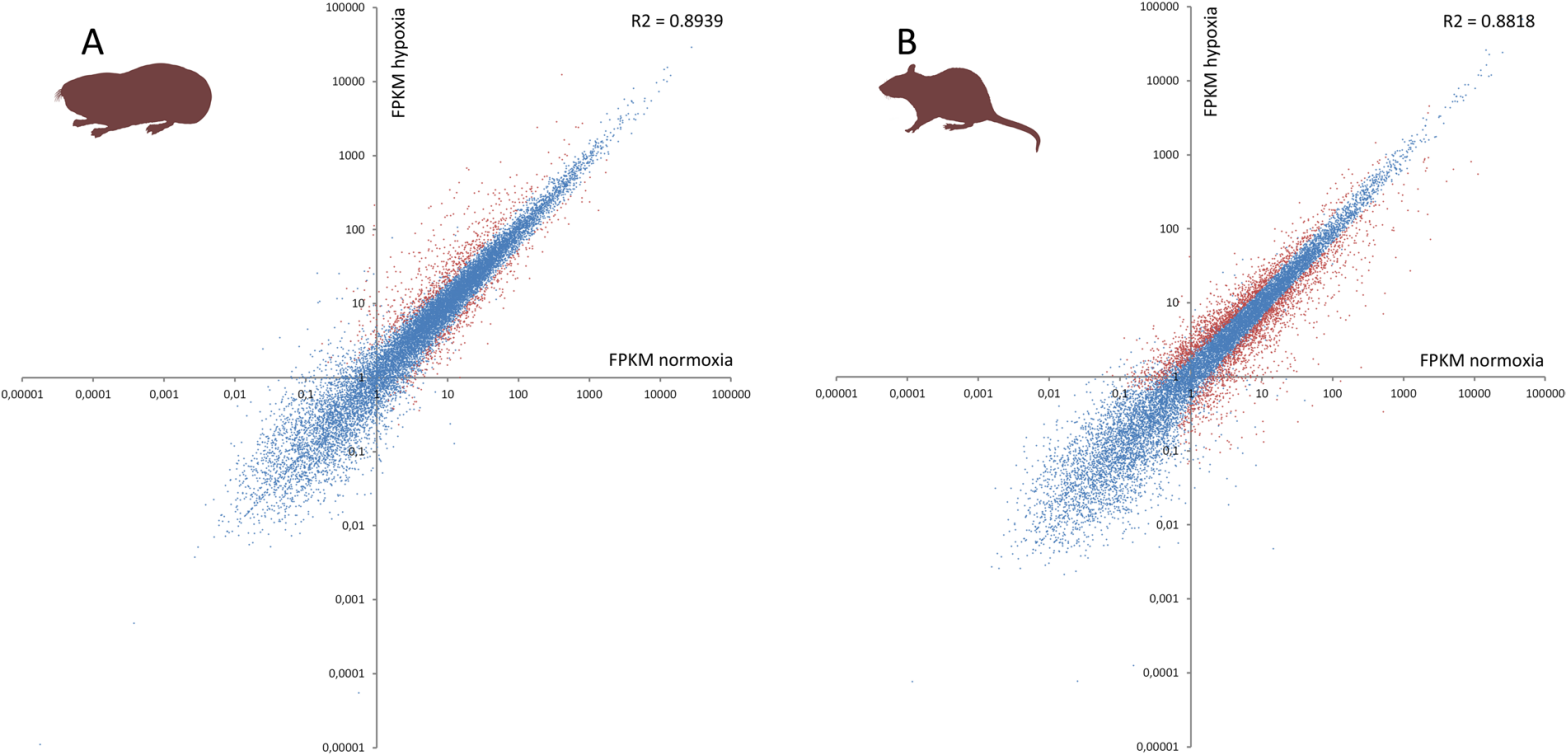
Differential gene expression in normoxia versus hypoxia measured in *Spalax* (**A**) and rat (**B**) The scatter-plot shows FPKM values for each measured gene (represented by a dot). Blue dots indicate genes not differentially regulated under hypoxic stress. Red dots represent genes with a significant gene expression change under hypoxia. A Cuffdiff q-value of 0.05 was set as the threshold. Note the logarithmic scales.

**Figure 2:**
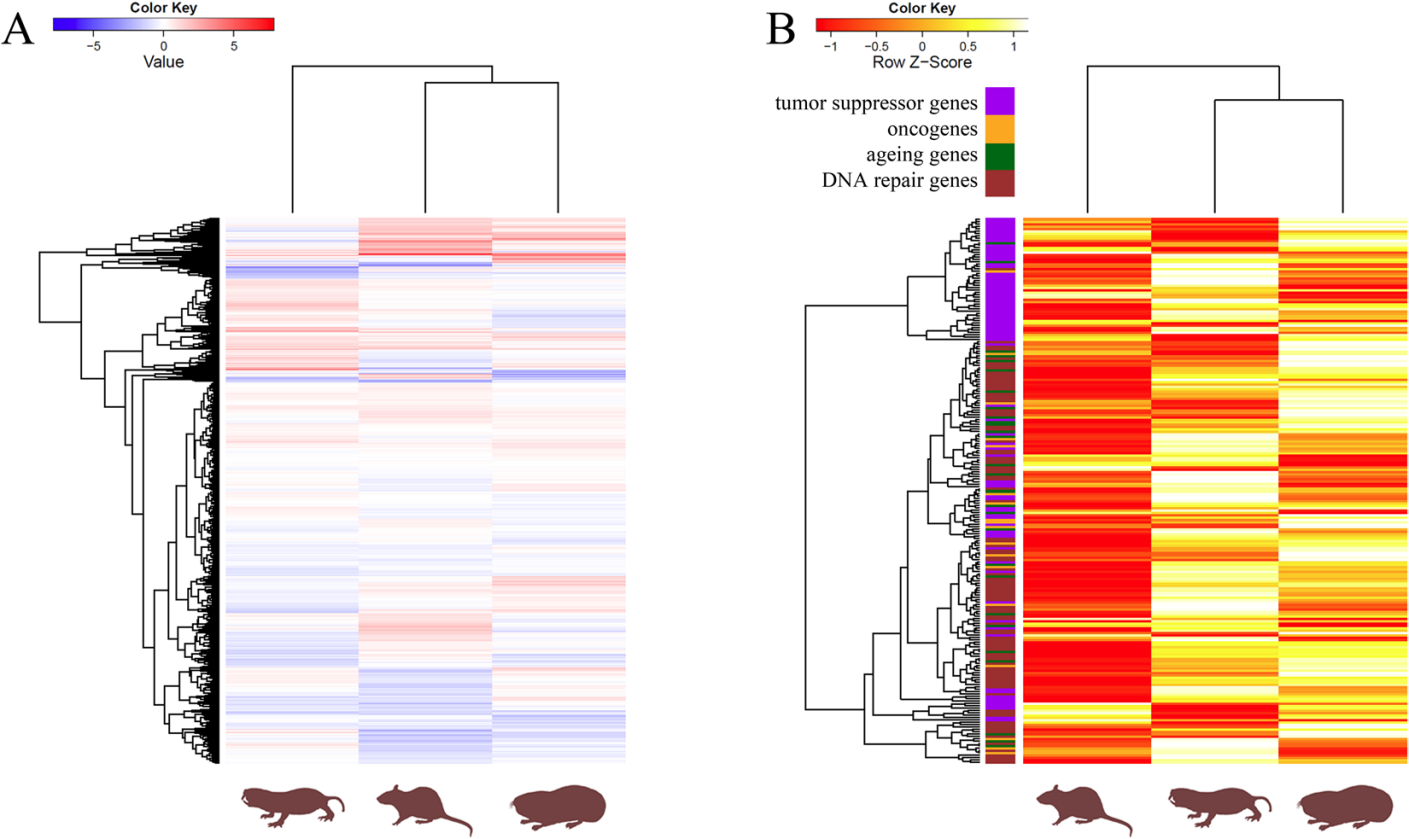
Gene expression heatmaps on a global scale (**A**) and for genes specifically involved in carcinogenesis, ageing and DNA repair (**B**) (**A**): Differential gene expression (log2-transformed fold changes) under hypoxia across the whole transcriptome. Red colour indicates up-, blue colour downregulation after hypoxic exposure. Species from the left to the right are: *Heterocephalus*, rat, *Spalax*. Note that the hypoxia response is globally different between the three species, with slightly more similarity between the phylogenetically closer related rat and *Spalax*. B: Level of transcriptional gene expression in genes involved in cancer, ageing and DNA repair processes (see text) at normoxia. The hotter the colour, the higher the gene expression value. The bar left to the species columns shows membership to the respective functional classes of genes. Species from the left to the right: rat, *Heterocephalus*, *Spalax*. The heat map pattern suggests a closer similarity of the two hypoxia-tolerant rodents. Note that the *Heterocephalus* data must be interpreted with caution since they originated from a single published dataset using a different hypoxia regime, thus prohibiting statistical verification.

The gene FPKM values for normoxic and hypoxic conditions correlated highly within the species (coefficient of determination of 0.89 in *Spalax* and 0.88 in rat across all genes) ((Supplementary dataset 1). We found 1,722 (12%) genes to be differentially expressed in *Spalax* between normoxic and hypoxic conditions, and 4,046 (26.9%) in rat (Fig. 1). In both species roughly three quarters (72.3% in *Spalax*, 74.8% in rat) of differentially expressed genes were up- and one quarter downregulated in response to hypoxia. In the *Heterocephalus* liver transcriptome data, 17,586 genes showed expression in at least one sample. Due to missing replicates in this taxon, we only could detect significance of hypoxia-induced differential expression for 39 genes. General pattern of hypoxia-induced gene regulation for all three rodents were highly species-specific on the transcriptome-wide scale and suggested only a slightly higher similarity in expression response between *Spalax* and rat, possibly reflecting their closer phylogenetic relationship (Fig. 2A).

### Pathway analysis of the hypoxia response

**Figure 3:**
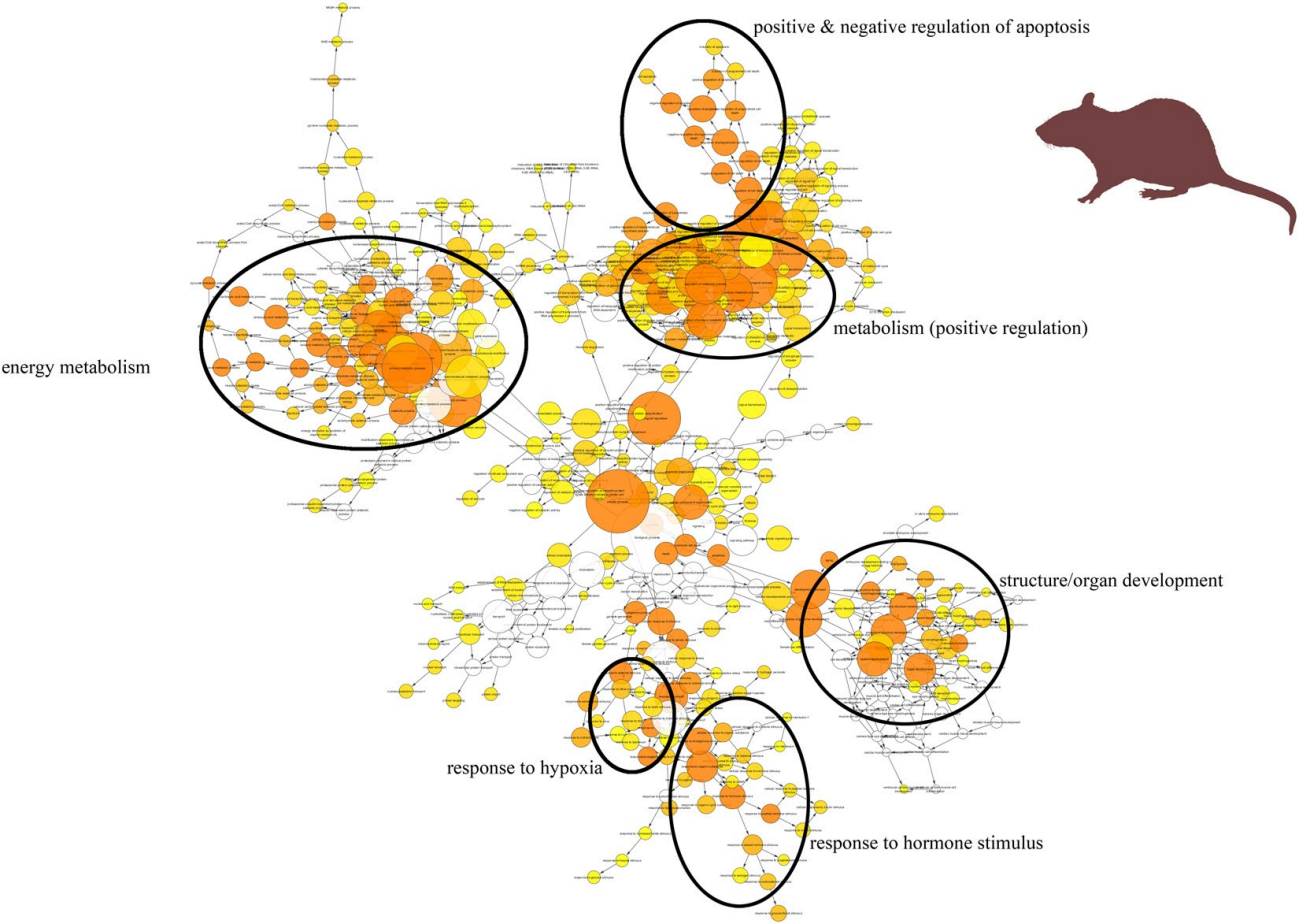
GO term cluster analysis for genes upregulated under hypoxia in rat Shown are GO terms from the category ‘Biological Process’ which show signs of significant enrichment amongst genes that are upregulated in rat under hypoxia, and their closest neighbours. Circles indicate clusters of GO terms linked to certain broader complexes. A darker colour represents a higher level of significance of enrichment.

**Figure 4:**
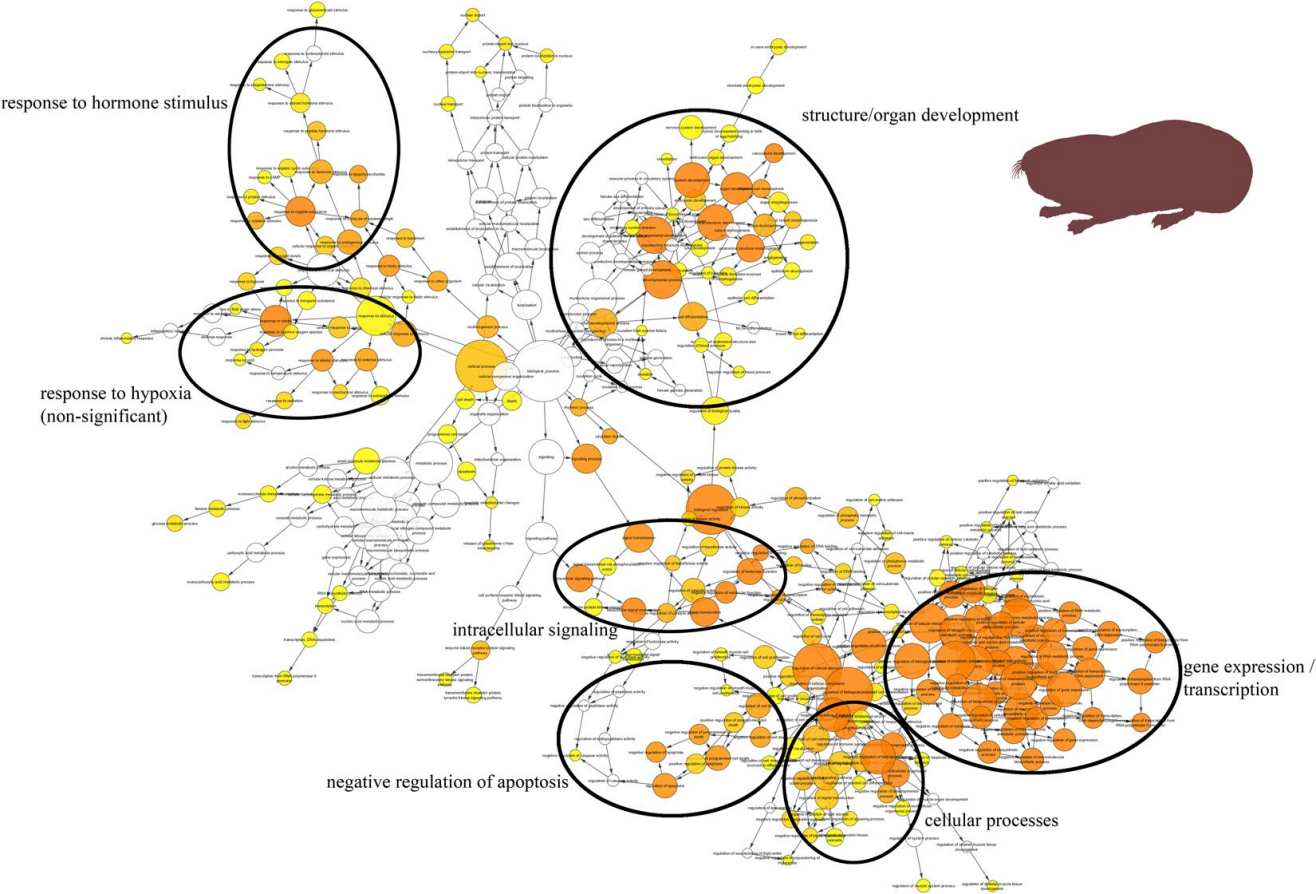
GO term cluster analysis for genes upregulated under hypoxia in *Spalax* Shown are GO terms from the category ‘Biological Process’ which show signs of significant enrichment amongst genes that are upregulated in *Spalax* under hypoxia, and their closest neighbours. Circles indicate clusters of GO terms linked to certain broader complexes. A darker colour represents a higher level of significance of enrichment.

The genes differentially regulated by induction of hypoxic stress were functionally annotated by assignment of gene ontology (GO) designations. Amongst those differentially regulated genes, we could identify several GO terms that were significantly enriched. *Spalax* and rat showed similar numbers of enriched GO terms for their genes upregulated under hypoxia (Table 1). In rat, the overall pattern of linked GO terms was represented by six clusters (Fig. 3). These clusters comprised GO terms associated with ‘positive regulation of metabolism’, ‘energy metabolism’, ‘response to hypoxia’, ‘structure/organ development’, ‘positive & negative regulation of apoptosis’ and ‘response to hormone stimulus’ (Fig. 3). The clusters ‘structure/organ development’, ‘response to hormone stimulus’ and ‘response to hypoxia´ also emerged in the graph of GO terms for *Spalax* (Fig. 4), the latter however not reaching statistical significance. In addition, *Spalax* showed GO term clusters linked to ‘intracellular signalling’, ‘negative regulation of apoptosis’, ‘cellular processes’ and ‘gene expression/transcription’, with the latter being the most prominent in terms of quantity. Of note, both species showed hardly any GO terms which were significantly enriched amongst the *down*regulated genes (Table 1). Some GO terms linked to ‘chemical sensory perception’ were underrepresented in the downregulated genes of *Spalax and* rat, and a few GO terms linked to ‘lipid biosynthetic processes’ were overrepresented in the genes only downregulated in rat.

KEGG pathway enrichment analysis of the hypoxia response revealed striking interspecific differences, mostly being associated with metabolism and signalling processes (Table 2). In the subset of rat hypoxia-upregulated genes, the term ‘metabolic pathway’ showed highest significance, followed by the related pathways ‘glycolysis/gluconeogenesis’ and ‘pentose phosphate pathway’. None of these was listed amongst the significantly enriched pathways in *Spalax*. In hypoxia-downregulated genes of *Spalax*, two of the most significant pathways were associated with cytochrome P450 activity. KEGG pathways enriched with *up*regulated genes in *Spalax* included ‘MAPK signaling’, ‘pathways in cancer’ and ‘circadian rhythm’, all of which are linked to cell cycle regulation (Table 2).

**Table 2:** KEGG pathway enrichment analysis of hypoxia-regulated genes.

***Spalax***		**rat**	
**upregulated**	**downregulated**	**upregulated**	**downregulated**
circadian rhythm	metabolic pathways	metabolic pathways	metabolic pathways
MAPK signaling pathway	metabolism of xenobiotics by cytochrome P450	RNA transport	RIG-I-like receptor signaling pathway
adipocytokine signaling pathway	drug metabolism - cytochrome P450	glycolysis/gluconeogenesis	terpenoid backbone biosynthesis
insulin signaling pathway	glutathione metabolism	ribosome biogenesis in eukaryotes	cytosolic DNA-sensing pathway
pathways in cancer	histidine metabolism	pentose phosphate pathway	Hepatitis C

Shown are the ‘Top Five’ KEGG pathways significantly enriched (p < 0.01) with significantly regulated genes (p < 0.01) in the two species, respectively.

### Species differences in *constitutive* gene expression associated with cancer, ageing, DNA repair and hypoxia

The GO analysis of the many genes more abundantly expressed in *Spalax* vs. rat under normoxia (i.e. constitutively without hypoxic stress exposure) yielded highly significant terms, which were all broadly associated with general cell metabolism (Supplementary dataset 3), thus making it difficult to infer more specific functional hypotheses. Genes higher expressed in rat were also mostly associated with the GO term ‘metabolism’, but at lower p-value significances. Interestingly, however, we detected a number of specific GO terms in the list of genes more abundantly expressed in *Spalax* (i.e. DNA damage, cell cycle, apoptosis) that were not enriched amongst genes expressed to a higher extent in rat. With the adaptive phenotype of *Spalax* in mind, this led us to specifically compare normoxic expression values of genes potentially involved in DNA repair, carcinogenesis and ageing between the rodent species. Broadly visualizing the strength of constitutive, normoxic expression of these functional gene categories, a high similarity between the two phylogenetically distant mole rats emerged from the heat-map picture (Fig. 2B). This similarity reflects the general finding that the majority of genes were expressed at higher transcript levels in both, *Spalax* and *Heterocephalus*, as opposed to rat (see below). Again have in mind that the *Heterocephalus* transcriptome data lacked replicates, so that interpretations below must be viewed with a certain caution.

Investigating functional gene categories, the majority of candidate oncogenes (15 of 24 genes; all having FPKM >0.1 in either species) were transcribed at ≥2-fold higher levels in *Spalax* than in rat at normoxia. Only two genes were expressed 2-fold higher in rat than in *Spalax* (Supplementary dataset 4). Only for one of the 15 genes (*MSH2*, a p53-targeted DNA repair gene), the augmented normoxic expression in *Spalax* was compensated for in rat by hypoxia-induced upregulation. A tendency towards increased normoxic expression in *Heterocephalus* was noted for 8 of the 15 *Spalax* cases. Out of 107 expressed candidate tumour-suppressors genes, 49 showed significant 2-fold higher mRNA levels in *Spalax* liver, while only 12 were higher expressed in rat (Supplementary dataset 4). In 4 genes (*GADD45B*, *BHMT*, *SDS*, *OTC*), rat compensated the lower normoxic expression by its hypoxia response. Of the 49 genes higher expressed in *Spalax*, 34 showed the same tendency in *Heterocephalus*.

Amongst genes linked to the ageing process, 28 of 48 were expressed at >2-fold higher levels in normoxic *Spalax* liver compared to rat (Supplementary dataset 5). Of those 28, 17 also showed the tendency for higher normoxic expression in *Heterocephalus*. Ten of these genes, namely *BUB3*, *ATR*, *A2M*, *HELLS*, *WRN*, *CISD2*, *CETP*, *SLC13A1*, *AGTR1* and *GH1*, were expressed more than 10-fold higher in normoxic *Spalax* than in rat. Not a single gene was overexpressed to such high extent in rat, where only five genes (*MSRA*, *ARNTL*, *PLAU*, *ADCY5*, *FGF21*) had FPKM values at least 2-fold higher than their *Spalax* counterparts. Interestingly, expression of the longevity-associated fibroblast growth factor 21 gene (*FGF21*) was approximately 10x lower in *Spalax* than rat; however, *FGF21* was 123-fold upregulated in *Spalax* by hypoxia, thereby out-numbering rat mRNA levels by 10-fold.

In our list of 167 genes associated with DNA damage and repair, 103 showed >2-fold higher expression in normoxic *Spalax* liver than in rat, and 19 genes were higher expressed in rat than *Spalax* (Supplementary dataset 6). Of those 103, 67 also showed elevated FPKM values in *Heterocephalus*. The remarkable number of 29 genes had >10-fold higher FPKM values in *Spalax* versus rat. Of special note, 18 genes from the Fanconi anemia (FA) DNA repair pathway were at least 2-fold and 12 genes even >10-fold upregulated in normoxic *Spalax* liver versus rat (labelled in Supplementary dataset 6). Involvement of this crucial pathway clearly points at differences amongst the two rodents in dealing with DNA damage and its repair. Additionally, Rae *et al*.^4^ have published a list of 34 DNA damage and repair genes, which showed significantly elevated expression in *Heterocephalus* liver compared to mouse. For 31 of those, we have obtained FPKM expression values from both *Spalax* and rat, and detected that 21 genes were >2-fold higher expressed in *Spalax* than rat.

Of n = 6 genes from the FOXO3 cell survival pathway, which were discussed with regard to their hypoxia-regulated differential expression in *Spalax* muscle (comp. Fig. 10 in Ref.^16^), 5 also showed hypoxia-regulation in *Spalax* liver (Supplementary dataset 7). From our extended list of hypoxia-related genes (n = 48; Supplementary dataset 7), 24 genes showed >2-fold and eight >10-fold higher FPKM values in normoxic *Spalax* liver than in rat, while 5 genes showed the opposite. Inspecting individual genes of known importance in hypoxia response, *HIF1A* showed a mild (2.4-fold) hypoxic transcriptional induction in *Spalax* liver and about equivalent normoxic FPKM levels in *Spalax* and rat. *Spalax* liver *HIF2A* mRNA levels exceeded those of *Spalax HIF1A* (9-fold) and those of rat *HIF2A* (12-fold). Vascular endothelial growth factor (*VEGFA*), a master factor of angiogenesis, was only slightly transcriptionally activated in *Spalax* and rat liver (1.5 and 1.8-fold). However, the anti-angiogenic factor *TSP1* showed 7-fold hypoxic upregulation only in *Spalax*.

An RNA-Seq study comparing liver tissue of mice and *Heterocephalus* reported the strongly increased expression of a set of mitochondrial and oxidation reduction genes in *Heterocephalus* ^26^. Amongst the set of genes tested in this study, alpha2-macroglobulin (*A2M*), a potential biomarker for ageing, showed a high increase of expression in *Heterocephalus*. Our data were strikingly in line, with an FPKM = 303 for *Spalax A2M*, but virtually no *A2M* transcription in rat (FPKM <0.01) (Supplementary dataset 8). Under hypoxia, *A2M* transcription was detectable in rat (FPKM = 15), but *Spalax* still overcompensated this (FPKM = 398).

### Validation of the RNA-Seq analysis

We complemented the comprehensive RNA-Seq analysis presented above by a second bioinformatic approach, seeking to eliminate potential biases resulting from the choice of methods. This approach differed in two aspects: (1) To particularly strengthen the interspecies comparison, an alternative bioinformatic pipeline producing very stringently defined *Spalax*-rat gene orthologs was applied, (2) different read-mapping and statistical validation algorithms, as well as other functional gene/pathway annotation tools were applied. Although these steps reduced the number of genes directly comparable between *Spalax* and rat to ca. 7,000, the major results of interspecies differential gene expression were confirmed and validated (Additional Note 1, Supplementary datasets 9–10).

In addition, we studied the hypoxia inducibility and interspecies expression differences of a selected set of important candidate genes of different functional categories (*A2M, ATR, CISD2, CITED2, FGF21, GNMT, PTEN, TOP2B, VEGFA, WRN, XPA*) by quantitative realtime RT-PCR (Additional Note 2). Thereby we could confirm the direction of all relevant gene expressional changes.

In order to account for potential biases in liver gene expression (sex, species, strain, age), we inferred transcriptome-wide FPKM values using public rat, mouse and human RNA-Seq data and compared them to our own data (Supplementary dataset 11). The results indicated that expression ratios, in particular those of females and males, did not exceed values of 2-fold for the vast majority of 200 candidate genes in rat, mouse and humans. Hence, such biases are improbable to substantially affect the interspecific and hypoxia-induced gene expression changes reported in our study.

### Signs of positive selection in gene coding sequences

In addition to gene expression changes, Darwinian positive selection for functional sequence changes may cause species-specific adaptations in *Spalax* genes. The coding sequences of 125 genes (Supplementary dataset 12), which were either classified as universal hypoxia genes showing signs of adaptation in other species^27^ or as functionally related to DNA repair and ageing, were therefore analysed for sequence signatures of positive selection. Using standard bioinformatical procedures to infer the ratio of non-synonymous to synonymous nucleotide substitutions (ω), none of the genes showed prominent signs of positive selection on the *Spalax* branch, typically defined as ω ≥ 1 (Supplementary dataset12). However, manual inspection of amino acid sequence alignments revealed several *Spalax*-specific substitutions, which nevertheless appear interesting and might require further attention. A conspicuous example is given by the protein kinase mTOR, which displays 17 amino acid replacements at positions which are fixed in all other mammals included in our study (Additional Fig. 2). Twelve of these replacement positions are clustered within the FAT (FRAP-ATM-TTRAP) domain, while the rest of the protein is highly conserved.

## Discussion

The rationale of the present study was to pinpoint the most important features of molecular adaptation in the hypoxia-tolerant, long-lived and cancer-resistant rodent *Spalax*, by performing a transcriptome-wide analysis of gene expression in comparison to the phylogenetically related, hypoxia-sensitive biomedical model species rat. We focused on analysing liver gene expression since the liver represents a highly active metabolic organ, crucial for survival, which should readily unveil interspecies differences. Measured RNA levels as a proxy of gene expression can explain two thirds of the protein abundance in mammalian cells^28^, thus forming an excellent guiding basis for subsequent molecular biological experiments. The main results of our comparative RNA-Seq analysis should be largely independent of technical biases, since they were validated using different ortholog gene annotation pipelines, read-mapping algorithms and tools to identify and functionally interpret differential gene expression. Potential biological biases (e.g. male/female) were also relatively small, compared to the gene expression differences observed between species or treatments.

Analysing differential gene expression in liver after hypoxic exposure of animals, we found more than twice as many genes being upregulated under hypoxia in rat (26.9%) than in *Spalax* (12%). The O_2_ level chosen for experimental hypoxia (6% O_2_) should not have been too mild for *Spalax* to provoke its full hypoxia stress response, since we applied a value below the lowest O_2_ content of 7.2%, which was measured experimentally in *Spalax* burrows in its natural habitat^15^. An alternative explanation is that, because *Spalax* is frequently confronted with hypoxia, its hypoxia stress response genes might already be expressed constitutively at levels required to avoid hypoxic damage. Indeed, *Spalax* showed higher constitutive, normoxic expression levels in many genes, which are functionally related to the investigated traits hypoxia, longevity, and tumorigenesis. The present comprehensive transcriptome comparison thus confirms and extends previous studies on phenotypically relevant candidate genes, which consistently displayed elevated expression in the blind mole rat (e.g. vascular endothelian growth factor^14^, hypoxia inducible factor and erythropoietin^15^, ROS defense genes^24^, globins^25^). Despite lacking replicate datasets and a different hypoxia regime, a similar trend of constitutively higher mRNA expression was also seen in naked mole rat liver for many genes involved in carcinogenesis, ageing and DNA damage repair. A noteworthy example is the increased expression of alpha-2 macroglobulin (*A2M*) in both mole rats, as opposed to rat and mouse (discussed below). With all necessary caution, this finding raises the interesting possibility that an augmented expression of genes has evolved convergently in the two distantly related, hypoxia-tolerant rodents. Constitutive, high expression of stress-response genes may confer a selective advantage to species encountering either extreme acute hypoxia (like *Spalax* at times of flooding in heavy soils) or chronic hypoxia (like *Heterocephalus* in its crowded eusocial colonies) by saving time and energy to switch-on the stress response. A hypoxia-sensitive species such as rat, which only occasionally encounters short-term mild hypoxia, may instead profit from a dedicated stress-induced gene regulatory response. Different strategies of gene regulation may thus have shaped the adaptive evolution of hypoxia-tolerant and -sensitive rodents. In contrast, our molecular evolutionary analysis of coding sequences revealed a lack of pronounced Darwinian evolution within candidate genes associated with ageing, longevity, and DNA repair and also in genes previously shown to be involved in hypoxia adaptation in humans and fruit flies^27^. Therefore, the *Spalax* phenotype might have evolved predominantly by gene regulatory changes, which in turn might have been orchestrated by positively selected key amino acid changes in important transcriptional master regulators like p53^19^ and the Nrf2-Keap1 complex^29^. Interestingly, here we report *Spalax*-specific amino acid replacements in the FAT domain of the mTOR protein (Additional Fig. 2), which is a key player in coupling cancer susceptibility, ageing and energy metabolism^30^. In addition, mTOR is involved in cell signalling and carcinogenesis^31^. Gain-of-function-mutations in the mTOR FAT domain cause hyperactivation, leading to increased proliferation of cancer cells and poor prognostic outcomes for patients^32^. The mTOR pathway in turn controls cell proliferation via activation of the FOXO3 transcription factor^33^. Indeed we found an hypoxia-induced upregulation of the FOXO3 pathway genes in the *Spalax* liver transcriptomes, in agreement with published data for *Spalax* myocytes^16^. We hypothesize that the increased transcription in the FOXO3 pathway might be caused by the mutated mTOR protein.

A general outcome emerging from our study is that both species, *Spalax* and rat, react to hypoxia by differential gene expression. However, the hypoxia-adapted *Spalax* regulates substantially less genes than the rat when temporarily exposed to 6% O_2_. In both species, the hypoxia-*up*regulated genes showed many more overrepresented GO terms than the *down*regulated genes (total gene numbers were approximately equal). Hypoxia thus prompted a strong *up*regulatory gene response, most probably by hypoxia-inducible transcriptional activators such as HIF1^34^.

In the hypoxia-induced genes of both rodents, GO terms linked to ‘structure/organ development’ and ‘response to hormone stimulus’ were enriched, in line with a generally known stress reaction of many organs. In addition, the enriched term ‘structure/organ development’ with its sub-terms ‘angiogenesis’ and ‘vasculature development’ implicates an initiation of vascularisation, possibly to ensure oxygen supply during hypoxic stress. Stimulation of vascularisation seemed to be more pronounced in rat, suggesting that *Spalax* livers are already well vascularized, as indeed shown before in *Spalax* muscle tissue^14^. Again, in both species GO terms linked to stress were identified. However, only in rat the specific GO cluster ‘response to hypoxia’ was significantly enriched in hypoxia-inducible genes, while in *Spalax* only the more general term ‘response to stress’ emerged. This is in line with the more pronounced hypoxia response of the rat, as noted before (Fig. 1).

Another enriched GO term cluster among hypoxia-regulated genes of both species was ‘regulation of apoptosis’. In rat, GO terms ‘positive regulation of apoptosis’ as well as ‘negative regulation of apoptosis’ were enriched, while in *Spalax* only the sub-branch ‘negative regulation of apoptosis’ appeared. This finding is in agreement with the genome-wide analysis of sequence evolution and gene duplication events in the apoptosis pathway of *Spalax* ^7^, which suggested a repression of apoptosis in *Spalax*, possibly to avoid cell loss during physiological stress.

Besides these shared expression patterns of hypoxia-upregulated genes, *Spalax* and rat also showed species-specific clusters of enriched GO terms. In rat, two major clusters were related to ‘positive regulation of metabolism’ and ‘energy metabolism’. The latter included GO terms like ‘catabolic process’ and ‘saccharide metabolic processes’. At the same time, GO terms linked to ‘lipid biosynthetic processes’ emerged amongst hypoxia-downregulated genes. Together, we conclude that the rat maintains its metabolism under heavy stress by catabolic processes. The KEGG pathway analysis confirmed this hypothesis: The hypoxia-sensitive rat markedly upregulated metabolic pathways including glycolysis/gluconeogenesis, a most critical metabolic function of the liver. Since this is part of a known common reaction to hepatic stress^35^, the observed gene regulation pattern can be seen as an attempt of a hypoxia-sensitive species to cope with the life-threatening stress of 6% O_2_. In stark contrast, *Spalax* showed *down*regulation of metabolic genes under hypoxia, including those involved in the p450 detoxification system playing a major role in liver. Downregulation of many energy-consuming processes in *Spalax* under acute hypoxia has previously been shown for brain tissues (see Supplementary Information in Ref^7^), highlighting the general importance of this strategy. It has well been recognized that a coordinated downregulation of biosynthetic, proliferative and energetic pathways is a hallmark of hypoxia/anoxia-tolerant vertebrates^36^. By preventing a lethal depletion in ATP, hypometabolism is considered a key adaptation to survive and recover from the lack of O_2_.

**Figure 5:**
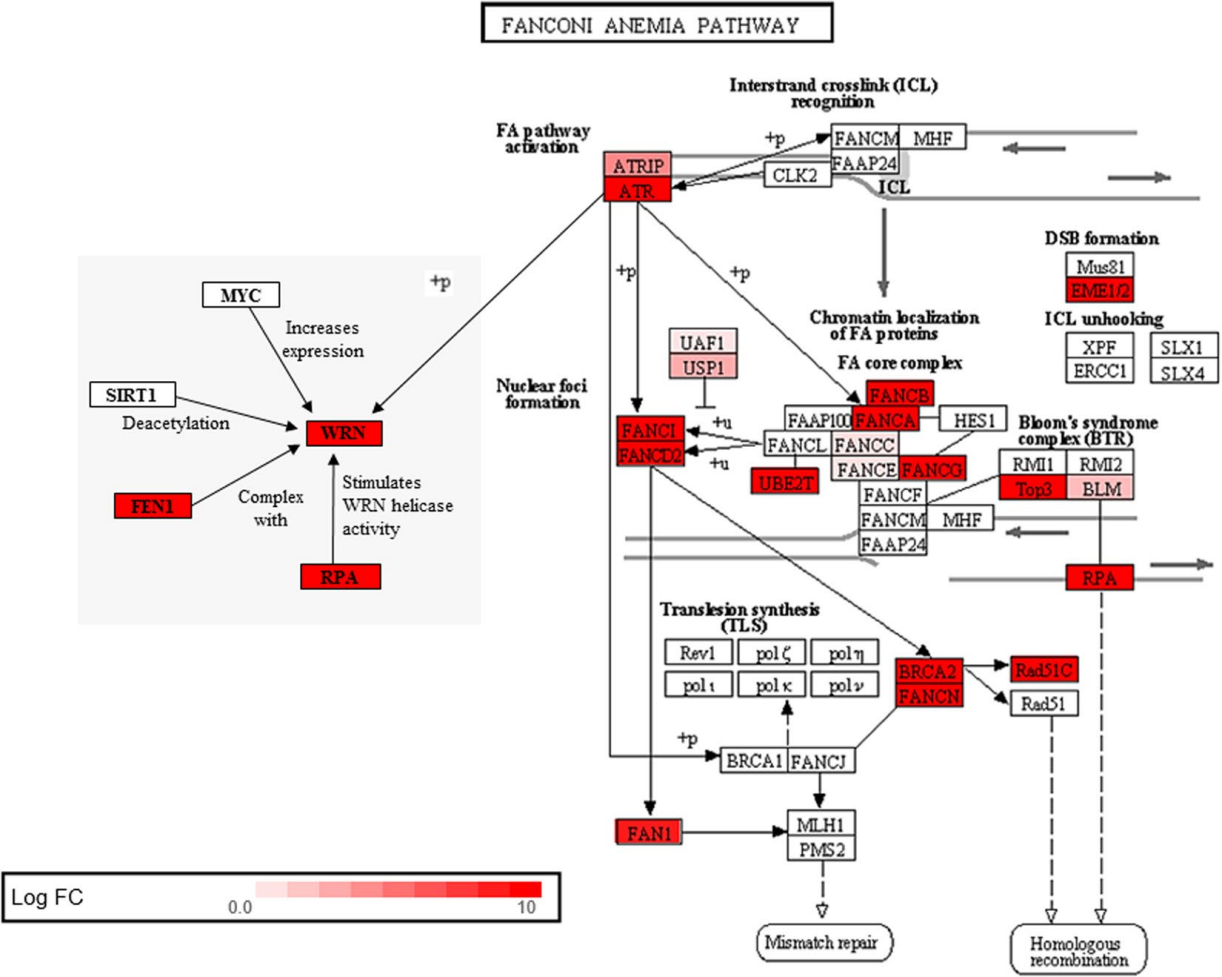
DNA damage and repair genes upregulated in *Spalax* liver as compared to rat. N = 18 genes from the Fanconi Anemia (FA) pathway were transcriptionally upregulated in normoxic *Spalax* liver, as indicated by the colour chart (prepared using Advaita Bio’s iPathwayGuide; http://www.advaitabio.com/ipathwayguide.html). Note that both, the major activator of this pathway, *ATR*, and its downstream interacting partner, the helicase *WRN* are overexpressed on the RNA level in *Spalax* versus rat liver. The same tendency was shown by *RPA*, which stimulates the helicase and exonuclease activity of WRN, and by FEN1, which complexes WRN to adjust stalled replication forks.

In addition to metabolic changes, *Spalax* liver genes upregulated under hypoxia were associated with roles in tumourigenesis. We found these genes enriched for GO terms and KEGG pathways linked to intracellular signalling and cell cycle regulation. An example is the FOXO3 pathway, which controls tumourigenesis^37^ and is moreover associated with longevity^38^. In parallel, the candidate anti-angiogenic gene *TSP1* was induced by hypoxia. Many of these gene regulatory changes in cancer- and angiogenesis-related genes were previously also observed in *Spalax* muscle and might contribute to a cancer-delimiting phenotype (comp.^16^). It will thus be important to investigate in detail if and how *Spalax* modulates these functions and possibly creates resilience to tumour formation as a by-product of its species-specific hypoxia response. Several additional candidate genes require special attention in this context:

- *BUB3*, transcribed 28-fold higher in *Spalax* liver than in rat, is essential for the spindle assembly checkpoint in higher eukaryotes. While a complete knockout of *BUB3* is lethal to mice already *in utero*, haplo-insufficiency leads to chromosome missegregation and increased susceptibility to chemical-induced tumourigenesis^39^. BUB3 further cooperates with the mitotic checkpoint protein RAE1. Mice double haplo-insufficient for *BUB3* and *RAE1*, but not single haplo-insufficient animals, produce a premature senescence phenotype^40^. Interestingly, also *RAE1* expression is augmented in *Spalax* versus rat (5.8-fold), and also *Heterocephalus* shows higher expression levels than rat for both genes (9.3-fold for *BUB3* and 4.1-fold for *RAE1*). Together, this indicates a potential impact of the BUB3/RAE1-complex on longevity and cancer resistance in both mole rat species.
- While expressed at low levels in normoxic *Spalax* liver, transcription of the liver-secreted hormone fibroblast growth factor 21 (*FGF21*) increased 123-fold under hypoxia. In humans, *FGF21* is upregulated during fasting and induces oxidative metabolism in peripheral tissues. Transgenic mice which overexpress *FGF21* showed increased lifespans^41^. FGF21 might thus be involved in linking the ecologically selected hypoxia response to the longevity phenotype.
- Another age-related gene, which is 12-fold overexpressed under normoxia in *Spalax* liver compared to rat, is the CDGSH Iron Sulfur Domain 2 (*CISD2*), a protein involved in cellular calcium homeostasis. Mutations of human *CISD2* cause Wolfram syndrome 2, a neurodegenerative disease associated with diabetes mellitus, optic atrophy and a decreased lifespan (OMIM 222300). *CISD2*-null mice showed early senescence and shortened life span compared to wildtype mice^42^. Interestingly, transgenic overexpression of *CISD2* in mice increased lifespan, possibly by an interconnection between calcium homeostasis and autophagy^43^.
- Longevity is plausibly associated with the activity of DNA damage repair pathways, which ensure genome integrity. Recent studies implied that long-lived species such as humans or *Heterocephalus* feature higher expression rates of DNA damage repair genes than short-lived organisms such as mice^4^. Brain tissue of the long-lived *Spalax* was also shown to display increased transcription of DNA damage and repair genes in comparison to rat^21^. We observed the same tendency in *Spalax* liver. For example, the DNA glycosylase *NEIL2* (involved in base excision repair) and the Xeroderma pigmentosum-associated *XPA* (a damage-specific DNA binding protein involved in nucleotide excision repair) are about 56- and 59-fold higher transcribed in normoxic *Spalax* liver than in rat. Overexpression of *XPA* in human cells increased their resistance to ultraviolet radiation-induced DNA damage^44,45^. However, more transgenic animal models or cell cultures with overexpressed repair genes, mimicking the natural gene expression pattern of mole rats, will be necessary to prove a direct correlation between increased levels of DNA damage and repair proteins and the longevity phenotype.
- Several genes associated with the Fanconi Anemia (FA) DNA repair pathway are expressed at much higher levels in *Spalax* than in rat (Fig. 5), including genes which encode the FA core complex and its main upstream activator ATR^46^. This essential pathway orchestrates recombination and repair processes which ensure chromosomal stability, while mutations in FA genes result in increased levels of chromosomal rearrangements and pre-dispose patients to cancer^47^. An intact FA pathway is required to prevent hypoxia-induced DNA damage in human cells *in vitro* ^48^. Overexpression of FA gene A (*FANCA*) decreased the susceptibility of human breast cancer cells to cross-linking agents^49^, and overexpression of a group C gene (*FAC*) protected mouse hematopoietic stem cells from apoptosis^50^. In line with increased expression of FA genes that could lead to an increased activity of DNA repair mechanisms, the *Spalax* genome also shows a reduced rate of chromosomal rearrangements^7^.

Interestingly, we also observed increased transcription in *Spalax* liver of the Werner syndrome helicase gene *WRN*, which encodes an interacting partner protein of the FA key activator ATR and functions as a helicase and exonuclease in many DNA repair processes^51,52^. Mutations in *WRN* cause the Werner syndrome, which is associated with premature ageing and an increased cancer susceptibility^53^. We assume that elevated expression levels of *ATR* and *WRN* might be linked to longevity and cancer resistance in *Spalax*, given that *Heterocephalus* also showed higher expression of these two genes. Recently, overexpression of *ATR* and *WRN* has been implicated in mediating autophagy, a cell-maintaining process known to be compromised in normal and pathological ageing^54,55^.

• The Alpha-2 macroglobulin (*A2M*) gene encodes a serum protease inhibitor produced by the liver. It has been found overexpressed in *Heterocephalus* compared to mouse^26^. Moreover, *Heterocephalus* plasma was described to contain higher A2M protein levels than human plasma^56^. Interestingly, our data also show increased *A2M* gene expression in *Spalax* compared to rat. A2M has been shown to have strong tumour-suppressor properties in astrocytoma cells, possibly by interfering with ß-catenin signalling^57^, and it was recently used to mediate tumour antigen presentation in cancer immunotherapy^58^. The convergence of high *A2M* expression in both mole rats highlights the protein as another promising candidate to further study its role in cancer resistance. In the presence of increased expression levels of highly potent tumour-suppressor and DNA repair genes, the observed increased expression of a subset of oncogenes in *Spalax* may be overcompensated for, thus leading to an overall suppression of tumourigenesis in this species.

We conclude that *Spalax* uses a completely different gene expression strategy to cope with hypoxic stress than rat in a vital tissue like liver. While the hypoxia-sensitive rat increases its cellular energy metabolism under hypoxia, a reaction possibly related to stress evasion, *Spalax* instead downregulates the majority of metabolic pathways. This latter response is indicative for the transition to a hypometabolic state and likely forms a crucial adaptation of *Spalax*, enabling its cells to survive strong, acute hypoxia without suffering from severe damage. Under hypoxia, *Spalax* differentially expresses genes involved in cell cycle regulation, hypothetically resulting in a tumourigenesis-controlling cellular state. A number of genes plausibly associated with cancer resistance, longevity and DNA damage repair are expressed at markedly higher levels in normoxic *Spalax* liver compared to rat, potentially ensuring constitutive protective effects. Altogether, transcriptome comparisons of hypoxia-tolerant versus -sensitive tissues of blind mole rats reveal multiple promising starting points for subsequent functional experiments in biomedical research and evolutionary genomics.

## Methods

### Animals, experiments and sequencing

All protocols of animal capturing, maintenance, experimental procedures and sacrificing were according to the regulations of the Israel Nature and Park Authority, Science and Conservation Unit, and have been approved by the Haifa University Committee on Animal Care. N = 6 *Spalax galili* (the northern subspecies of the four residing in Israel; female individuals captured from basalt, heavy soil) were used. The *Spalax* specimen were caught in the field and kept under routine veterinary scrutiny in the Animal House of the Institute of Evolution at Haifa University until approximately 4 years old (adults, 150–250 g). N = 6 *Rattus norvegicus* specimen (Sprague Dawley, 4 month old males, 350–400 g) were used. The age of the two species roughly corresponds given their different overall life expectancy. For the experiment, n = 3 specimen of *Spalax* and rats were kept under normoxic (21% O_2_) and n = 3 under hypoxic (6% O_2_) conditions for six hours each. This regime was chosen to ensure that a hypoxia response was elicited. No time-series of hypoxic gene expression exist for *Spalax* liver, but mRNA quantification in *Spalax* kidney indicated that HIF1a mRNA markedly increased after 4 hours (6% O_2_) and stayed high until 24 hours (10% O_2_)^59^. Evidence for a successful hypoxia response is indicated by upregulation of known HIF1a target genes (see Supplementary dataset 7).

For hypoxia exposure, we used 70 × 70 × 50 cm chambers divided into separate cells and a gas flow rate of 3.5 l/min. All animals were adults of similar weight (~150 g). After the six hours of exposure, animals were immediately sacrificed by injection with Ketaset CIII (Fort Dodge, USA) at 5 mg/kg of body weight and their tissues harvested and transferred to liquid nitrogen. Rat specimen were kept and processed under the same conditions. RNA samples from the liver right lobe of twelve animals was extracted with Qiagen RNeasy Mini Kit (Qiagen, Germany) including an on-column DNase I digestion and quality checked with a RNA nanochip on an Agilent 2100 Bioanalyzer (Agilent Technologies, CA, USA). Libraries for high-throughput cDNA sequencing (RNA-Seq) were constructed using the TrueSeq RNA Sample Prep Kit v.2 (Illumina, CA, USA) tagged with different multiplex indices (StarSEQ, Mainz, Germany). All six libraries were sequenced as 100 bp paired-end reads on an Illumina HiSeq 2500 instrument (Institute of Organismic and Molecular Evolution, University of Mainz, Germany).

### Raw data processing

All reads from the 12 samples were processed prior to analyses using the FASTX toolkit 0.0.13 (http://hannonlab.cshl.edu/fastx_toolkit) with the following parameters: 15 bp cut from the 5′-end, removal of universal and index adapters, trimming of all positions at the reads’ ends with quality scores below 20, removal of all reads with less than 80% positions with a quality score of 20 or higher. All datasets were quality-checked with the FastQC software (http://www.bioinformatics.babraham.ac.uk/projects/fastqc/) multiple times to assure optimal results. For further interspecific comparison we downloaded RNA-Seq-derived liver transcriptome sequences of *Heterocephalus glaber* from NCBI’s Sequence Read Archive (SRA). One dataset was from an animal kept under normoxic conditions (SRA accession number SRR306395) and one from a hypoxia exposed individual (8% O_2_ for one week, SRR306404). The reads from these two datasets were processed in the same way as described above.

### Mapping and differential gene expression analysis

Reference genome sequences were downloaded from public databases, using version 1.0 of *Spalax’* genome from NCBI’s genome database and Ensemble release 72 for the rat genome. *Spalax* and rat RNA-Seq reads were mapped and analysed for differential expression (DE) using the TopHat-Cufflinks-Cuffmerge-Cuffquant-Cuffdiff-pipeline^60^. TopHat 2.0.11^61^ with Bowtie2 2.2.1^62^ was used for mapping and Cufflinks 2.1.1^63^ for DE analysis. A q-value of <0.05 was set as threshold for DE between normoxic and hypoxic triplicates for *Spalax* and rat FPKM values, respectively. Additionally, we calculated FPKM-fold-changes from normoxia to hypoxia, log2-transformed and then fed them into the heatmap.2-function from the R gplots package in RStudio 0.98.1060 (http://www.rstudio.com/).

### Gene ontology and pathway analyses

We used the vocabulary of the Gene Ontology (GO) consortium^64^ for annotation of genes, pathways and processes regulated under hypoxic conditions. GO enrichment analyses in the category ‘Biological Process’ were performed using the BiNGO 2.44 plugin^65^ for Cytoscape 3.1.1^66^, applying default parameters. A significance level of 0.01 was set as threshold for the Benjamini-Hochberg false-discovery rate-corrected hypergeometric test and *R. norvegicus* as the annotation background. Input lists consisted of significantly regulated genes (q-value < 0.01) for each of the two species, split into up- and downregulated genes. Each group of genes was checked for over- and under-representation of GO terms separately.

Additionally, we performed a search for KEGG pathways^67,68^ enriched with genes regulated under hypoxic conditions. For this task we used the webserver version of WebGestalt^69,70^ with *R. norvegicus* as the annotation background and a significance level of 0.01 for the Benjamini-Hochberg adjusted p-value.

### Compilation of genes related to cancer, ageing, DNA damage and repair and hypoxia

To compare expression differences between *Spalax* and rat in genes known to be differentially expressed in liver cancer, we created two sets of genes using the RNA Seq Atlas resource (http://medicalgenomics.org/rna_seq_atlas ^71^). The first list consisted of 32 genes consistently upregulated in 15 different liver cancer cell lines, representing candidate oncogenes. The second list consisted of 162 genes consistently downregulated in these 15 different liver cancer cell lines, i.e. candidate tumour-suppressor genes. We then compared the interspecific differences in the gene expression levels under normoxia and their regulation under hypoxia. A list of 54 genes with published links to the ageing process in humans (as studied by mammalian models) was downloaded from the database GenAge^72,73^ and complemented with literature knowledge to identify ageing-related genes differentially expressed between *Spalax* and rat. Since DNA repair mechanisms are important players in both, carcinogenesis and ageing, we additionally checked a list of relevant genes (n = 202 genes) derived from www.sciencepark.mdanderson.org and extracted from literature. A list of hypoxia-related genes (n = 60) was compiled to include the hypoxia-regulated FOXO3A pathway (regulated in *Spalax* muscle^16^), a list of genes involved in hypoxia adaptation in humans and *Drosophila* ^27^ and known hypoxia-responsive genes from literature. Besides manual inspection, the log2-transformed FPKM values under normoxia from the listed genes were fed into RStudio as described above with row scaling applied.

### Search for signs of positive selection

To additionally investigate the impact of positive selection within gene coding sequences as a candidate mechanism for the peculiar adaptations of *Spalax*, a subset of 125 ageing-, cancer- and hypoxia-associated genes (Supplementary dataset12) was screened on the sequence level in a phylogenetic context. Orthologous transcript sequences of candidate genes from *Spalax*, other rodents and outgroup mammals were downloaded from public databases and aligned using the MUSCLE algorithm^74^ implemented in MEGA 5.01^75^ with default settings. Alignments were trimmed to the coding sequence, manually inspected and corrected, and then used to carry out branch-specific tests of positive selection implemented in *codeml* as part of the program package PAML 4.9a^76,77^. Likelihood values from free ratio model tests were compared to likelihood values from one-ratio model tests to identify significant signs of positive selection on the *Spalax* branch. The underlying rodent phylogeny was obtained from Voloch *et al*.^78^.

### Data availability

The datasets supporting the conclusions of this article are available in the European Nucleotide Archive ENA under the study accession number PRJEB17935.

## Electronic supplementary material

Additional Information

Supplementary dataset 1

Supplementary dataset 2

Supplementary dataset 3

Supplementary dataset 4

Supplementary dataset 5

Supplementary dataset 6

Supplementary dataset 7

Supplementary dataset 8

Supplementary dataset 9

Supplementary dataset 10

Supplementary dataset 11

Supplementary dataset 12
