# Supplementary material for: Hypoxia tolerance, longevity and cancer-resistance in the mole rat Spalax – a liver transcriptomics approach
Source: Sci Rep. 2017 Oct 30;7:14348. doi: 10.1038/s41598-017-13905-z (PMC5662568; doi:10.1038/s41598-017-13905-z)
Supplement: Supplementary file 1 — Additional Information [file 41598_2017_13905_MOESM1_ESM.pdf]

Hypoxia tolerance, longevity and cancer-resistance in the mole rat *Spalax* – a liver transcriptomics approach

Hanno Schmidt, Assaf Malik, Anne Bicker, Gesa Poetzsch, Aaron Avivi, Imad Shams & Thomas Hankeln

***Scientific Reports* 2017**

Additional Information

Additional Table 1 - Number of reads for all sequencing libraries before and after trimming

Species	Sample	Raw read count	Processed read count
Spalax	normoxia 1	54,741,206	51,735,511
	normoxia 2	33,083,526	29,655,666
	normoxia 3	46,988,870	44,529,584
	hypoxia 1	53,275,036	50,698,645
	hypoxia 2	36,919,088	34,750,337
	hypoxia 3	31,988,836	28,565,218
rat	normoxia 1	51,359,068	47,693,277
	normoxia 2	43,230,350	40,211,676
	normoxia 3	58,087,930	53,798,675
	hypoxia 1	78,731,484	73,193,698
	hypoxia 2	41,762,988	38,789,952
	hypoxia 3	53,451,370	49,562,358
Heterocephalus	normoxia (SRR306395)	54,531,044	52,695,779
	hypoxia (SRR306404)	66,664,902	64,567,190

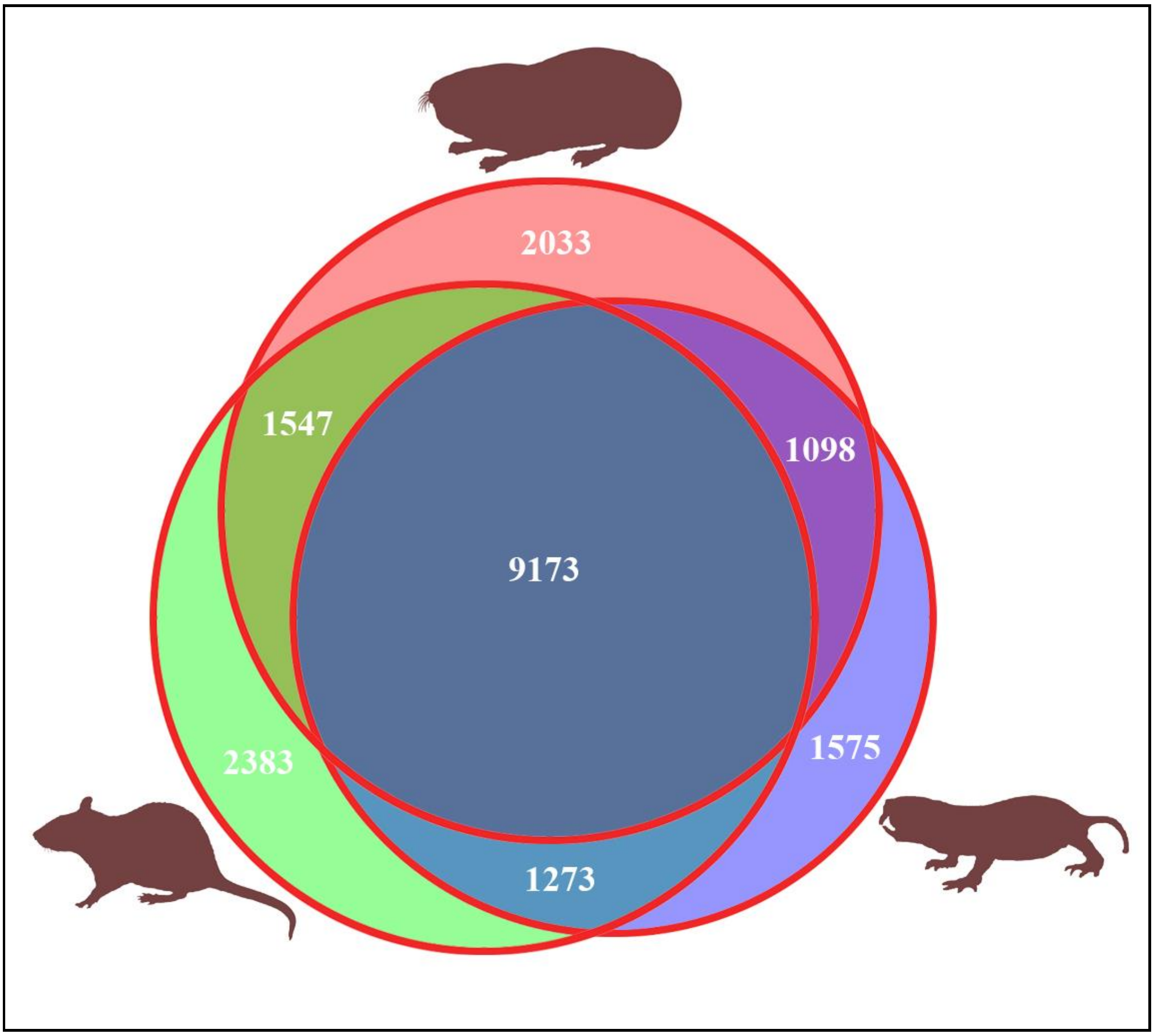

Additional Figure 1 - Venn diagram of the number of expressed genes

Shown are genes with detectable expression in *Spalax* (upper circle), rat (left circle) and *Heterocephalus* (right circle), and the overlap between the species.

Additional Note 1: Validation of the RNA-Seq analysis by alternative bioinformatics tools

To reassess the main results of our study, we selected a controlled set of gene orthologs for the interspecies comparison, thereby addressing specific issues in RNA-Seq analysis[1]. We show that this approach corroborates the main results.

Spalax vs. rat putative orthologous genes (1:1 orthologs only) were determined by Orthofinder[2] using rat Ensembl protein sequences (www.ensembl.org) and *Spalax* protein sequences (ftp://ftp.ncbi.nlm.nih.gov). We then prepared cross-species genomic annotation data for *Spalax* vs. rat. This was done as follows: (1) pair-wise alignments of i orthologous transcripts (i=13,000) were built using MAFFT v7[3,4]; (2) every alignment, a_i, was divided into j 25 bp sub-alignments; (3) for each sub-alignment, a_{ij}, with > 70% identity and gaps < 3 bp, the matching genomic regions $g_{ij,species1}$ and $g_{ij,species2}$ were retrieved and stored in gtf format; (4) for each gene, RNA-Seq coverage levels in all $g_{i,species1}$ and $g_{i,species2}$ genomic sub-regions were calculated using FeatureCounts[5]; (5) we excluded orthologous genes whose coverage along the gene was poorly correlated between the species ($r < 0.5$), unless the coverage was consistently higher in one species compared to the other (sign test P-value < 0.001, using R binom-test function); (6) for each gene i, we fitted all j sub-alignments in $g_{i,species1}$ vs. $g_{i,species2}$ to a linear regression model (RLM module in R), since we observed that this model correctly predicts reads coverage ratios between samples of the same species. RLM outliers were excluded, unless the sign test was significant (see previous step); (7) two final coordinates-files in gtf format were produced, one for each species, after excluding the above-mentioned incomparable genes and gene regions. Coverage plots were visually inspected using the IGV genome viewer and visualization scripts. The pipeline yielded 7,184 *Spalax*-rat 1:1 orthologous genes. We normalized the gene counts of all samples using EdgeR TMM method[6] and DESeq2 default normalization[7] and inferred differentially expressed genes (parameters: log_2 fold-change > 1.0, adj. p-value < 0.05).

<u>Clustering gene orthologs by species and treatment factors:</u> Based on the normalized read-count data of all comparable *Spalax*-rat orthologs over the twelve tested samples, multidimensional scaling (MDS) clustering clearly approved that the principal factors that govern the samples' similarity are the species identity, and the level of O_2 (Fig. A11-1). As the MDS shows, the species and the O_2 level effects explain about 80% and 10% of the variation,

respectively. This reflects the numbers and ratios of differentially expressed genes obtained by the analysis as presented in the main manuscript text.

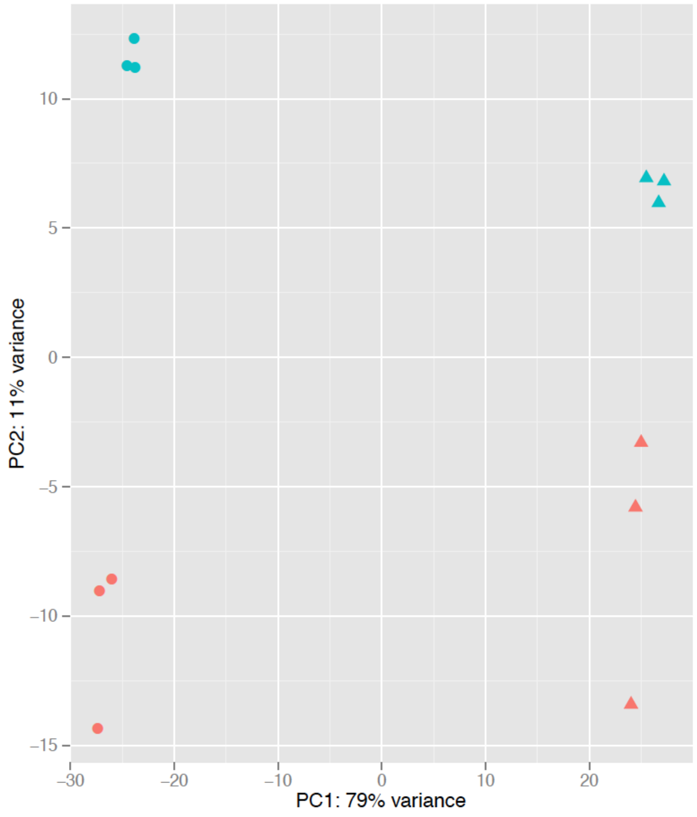

Fig. AN1-1: Interspecies multidimensional scaling (MDS) clustering

Principal component 1 (PC1) refers to the interspecies comparison, PC2 to the hypoxia-normoxia comparison (circles: rat, triangles: *Spalax*, blue: hypoxia, red: normoxia). This representation demonstrates the effect of both the species and the O_2-level factors on the transcript abundance.

<u>Functional enrichment among rat vs. *Spalax* differentially expressed genes:</u> We performed three differential expression (DE) comparisons: 1) all six rat individuals against all six *Spalax* individuals, where EdgeR GLM was used to test the additive effect of the species factor, the O_2-level factor, and the species × treatment interaction, on transcript abundance; 2) within the hypoxia group we compared three rat individuals against three *Spalax* individuals; 3) within the normoxia group, we also compared three rat individuals against three *Spalax* individuals. Significantly DE genes were defined by fold change > 2 or < -2 with a cutoff of 0.05 for the adjusted p-value. In each of the above three groups we identified significantly enriched functional groups among DE genes using ClusterProfiler[8]. Supplementary dataset 9 shows all significantly enriched terms and their gene symbols, Tab. A11-1 represent a

selected group of functional group of terms. A significant enrichment of metabolic genes is seen in all three comparisons among genes, which are higher expressed in rat than in *Spalax* (Tab. A11-1). Functional groups associated with lipid metabolism are significantly enriched in all three comparisons. On the other hand, metabolic terms associated with the mitochondrial respiratory chain complexes activity, and the wider functional group of oxidation/reduction and NAD/NADH metabolism, are significantly enriched only among genes higher expressed in rat vs. *Spalax* at hypoxia, but not at normoxia. This indicates that in hypoxia, large interspecies differences emerge within groups of genes that control ATP and ROS production, supporting the results summarized in Figs. 3 and 4 of the main manuscript. The observed differences in lipid metabolic pathways may be associated with the regulation of bioenergetics under hypoxia, for example via control of gluconeogenesis.

In addition, repair genes, DNA metabolism genes, and specifically Fanconi Anemia pathway genes (Fig. A11-2), which are critical for the response to DNA damage, are upregulated in *Spalax* compared to rat (Tab. A11-1), again confirming the main text conclusions (Figs. 2B and 5). These results indicate generic *Spalax* vs. rat differences in the expression patterns of genes associated with responses to stress and hypoxia.

Tab. AN1-1: functional enrichment among rat vs. *Spalax* differentially expressed genes

Functionally enriched groups among *Spalax* vs. rat up- or down-regulated genes (FC >2, adj. p value < 0.05); Column *in hyp + norm*: adj. *p* values of enrichment based on the comparison of all six rat individuals against all six *Spalax* individuals (in each group, individuals within both the hypoxia and the normoxia groups were used); Blank cells represent non-significant results. Column *in hyp*: adj. *p* values of enrichment based on the comparison of three rat individuals against three *Spalax* individuals, within the hypoxia group; Column "in norm": adj. *p* values of enrichment based on the comparison of three rat individuals against three *Spalax* individuals, within the normoxia group; Column *Functional terms*: functionally similar terms are shown in different colours. Analysis was done using clusterProfiler.

Rat vs. *Spalax* DE	functional term	#DE	adj. p value		
			in hyp + norm	in hyp	in norm
Spalax > rat, FC > 2, adj. p value < 0.05	cellular response to stimulus	490	7.8E-05	3.3E-04	3.7E-04
	signaling	393	1.6E-04	8.5E-03	1.2E-04
	DNA repair	62	4.8E-03		
	DNA metabolic process	100			9.3E-03
	Fanconi anemia pathway	19	1.8E-04	4.1E-04	1.4E-02
Spalax < rat, FC > 2, adj. p value < 0.05	oxidation-reduction process	117	2.4E-06	1.7E-06	
	lipid localization	50	3.1E-04	1.6E-03	9.9E-05
	oxoacid metabolic process	111	5.5E-04	3.1E-03	
	lipid transport	43	9.7E-04	6.6E-03	6.7E-05
	system development	312	5.2E-03	9.7E-03	5.1E-03
	cellular lipid metabolic process	95	6.0E-03		3.5E-03
	mitochondrial protein complex	28	1.7E-04	5.4E-04	
	mitochondrial envelope	82	2.7E-04	5.4E-04	
	mitochondrial part	101	1.3E-03	1.7E-03	
	inner mitochondrial membrane protein complex	19	3.1E-03		
	oxidoreductase complex	19	7.7E-03		
	respiratory chain complex	14	9.6E-03		
	mitochondrial respiratory chain complex I	11	1.0E-02		
	NADH dehydrogenase complex	11	1.0E-02		
	oxidoreductase activity	90	5.2E-07	4.8E-07	
	NAD binding	16	3.7E-03	2.2E-03	
	Parkinson's disease	23	4.4E-03	6.9E-03	
	Metabolic pathways	148	3.9E-02	2.3E-02	
	lipid biosynthetic process	65	2.4E-03		1.6E-03
	lipid metabolic process	115	3.3E-03		8.0E-03
	response to chemical	289	5.8E-04	5.5E-03	1.4E-05
	response to external stimulus	186	7.3E-04	5.7E-03	1.8E-06
	metabolic process	785	9.5E-04	5.5E-03	4.5E-05
	blood vessel morphogenesis	62	1.5E-03	9.3E-03	4.6E-03
	wound healing	57	1.1E-04	1.4E-03	6.2E-05
	cell communication	379	4.5E-04	5.5E-03	1.9E-04
	cell adhesion	108	4.7E-03		5.1E-03
	angiogenesis	52	4.8E-03		7.2E-03
	response to stress	286	5.8E-03		1.0E-04
	blood vessel development	65	7.0E-03		1.0E-02
	extracellular region	342	3.6E-07	2.8E-07	4.3E-05
	heparin binding	20	5.4E-03	9.4E-03	

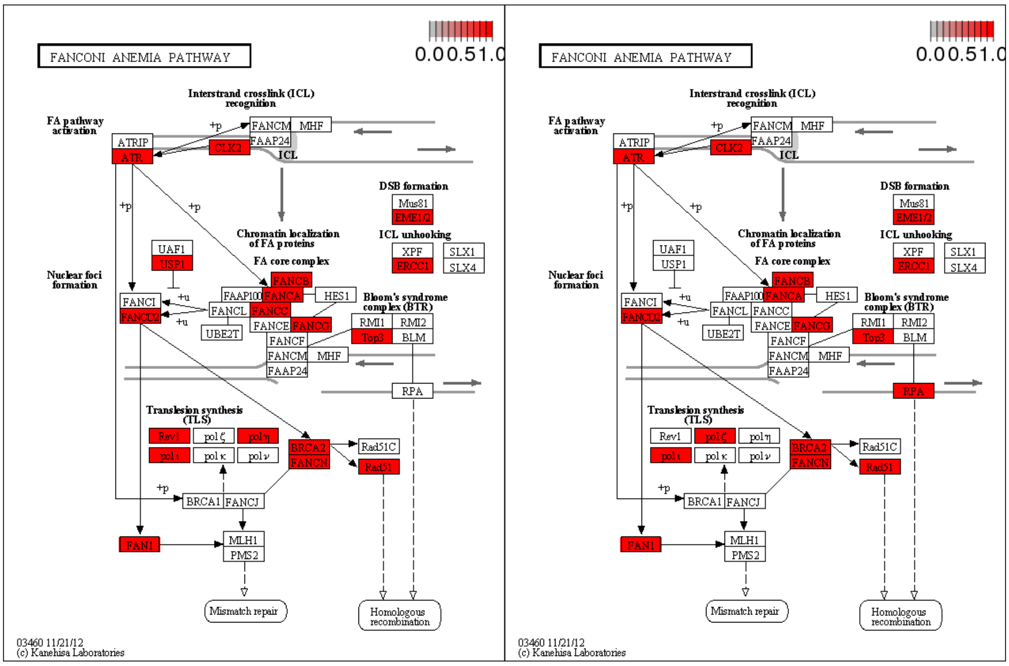

Fig. AN1-2: Genes significantly higher expressed by at least two fold in *Spalax* compared to rat within the significantly enriched Fanconi Anemia functional group

A large overlap exists between genes with significant > 2 fold expression differences in both hypoxia (right) and normoxia (left).

__**Functional enrichment among hypoxia responsive genes in *Spalax* and rat:**__ Using the controlled gene set, hypoxia-induced differential gene regulation was inferred separately for *Spalax* and rat, and significant functional categories of GO terms and KEGG pathways were identified (Supplementary dataset 10). In *Spalax*, these included the liver insulin resistance pathway (Fig. A11-3), and the Foxo signalling pathway (Fig. A11-4), again confirming results from the main text analysis. Both pathways can indeed be functionally interconnected, since the Foxo signalling pathway can be regulated by insulin.

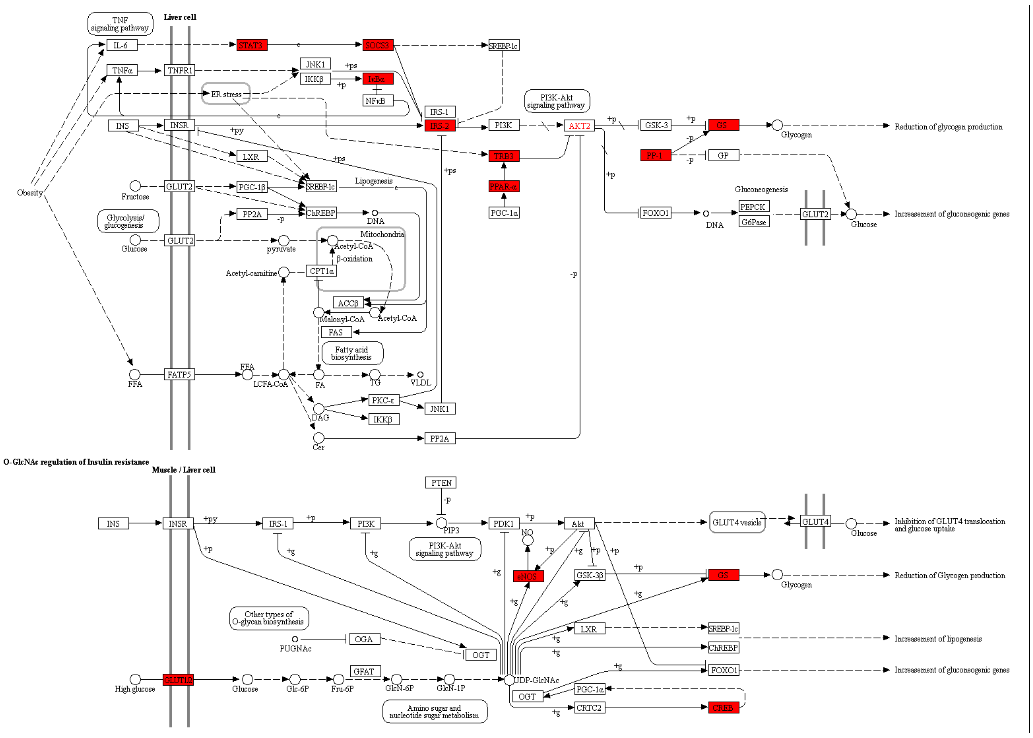

Fig. AN1-3: Genes of the liver insulin resistance pathway significantly upregulated at least two fold under hypoxia in *Spalax*

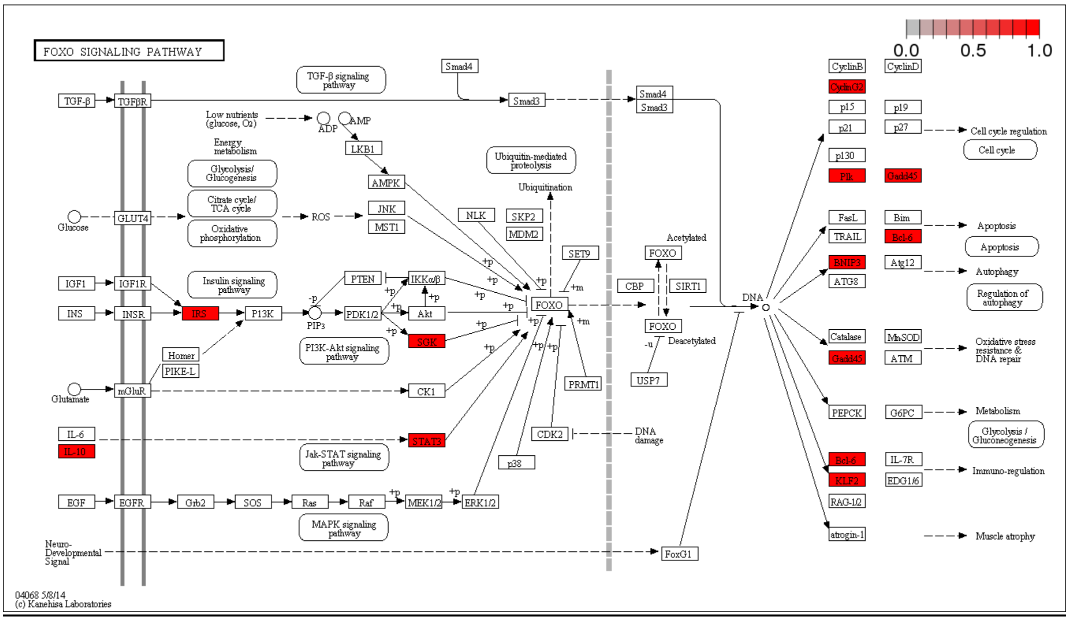

Fig. AN1-4: Genes of the FOXO signalling pathway significantly upregulated at least two fold under hypoxia in *Spalax*

References

1 Zhu, Y., Li, M., Sousa, A. M. M. & Šestan, N. XSAnno: a framework for building ortholog models in cross-species transcriptome comparisons. *BMC Genomics* **15**, 343 (2014).

2 Koren, S., Walenz, B. P., Berlin, K., Miller, J. R. & Phillippy, A. M. Canu: scalable and accurate long-read assembly via adaptive k-mer weighting and repeat separation. *bioRxiv*, doi: http://dx.doi.org/10.1101/071282 (2016).

3 Boetzer, M. & Pirovano, W. Toward almost closed genomes with GapFiller. *Genome Biol.* **13**, 1 (2012).

4 McKenna, A. *et al.* The Genome Analysis Toolkit: a MapReduce framework for analyzing next-generation DNA sequencing data. *Genome Res.* **20**, 1297-1303 (2010).

5 Li, H. *et al.* The sequence alignment/map format and SAMtools. *Bioinformatics* **25**, 2078-2079 (2009).

6 Robinson, M. D., McCarthy, D. J. & Smyth, G. K. edgeR: a Bioconductor package for differential expression analysis of digital gene expression data. *Bioinformatics* **26**, 139-140 (2010).

7 Auton, A. *et al.* Genetic recombination is targeted towards gene promoter regions in dogs. *PLoS Genet.* **9**, e1003984 (2013).

8 Browning, S. R. & Browning, B. L. Rapid and accurate haplotype phasing and missing-data inference for whole-genome association studies by use of localized haplotype clustering. *Am. J. Hum. Genet.* **81**, 1084-1097 (2007).

Additional Note 2: Validation of RNA-Seq data by qRT-PCR

To validate the differential expression of candidate genes observed by RNA-Seq in normoxic and hypoxic rat and *Spalax* liver samples, we performed quantitative realtime reverse-transcriptase PCR (qRT-PCR). First strand cDNA was synthesised from 600 ng of each RNA sample with the Superscript III RT kit (Thermo Fischer). For measuring expression, we chose absolute quantification by the standard curve approach and normalised expression on rigorously determined total RNA concentration. This method is especially recommended for comparing conditions in which reference genes are often misleading[1]. To construct standard curve plasmids, we generated PCR amplicons for each gene with the TrueStart Taq DNA Polymerase kit (Thermo Fischer) in a peqSTAR 96 Universal Gradient Thermocycler (Peqlab). Primers are listed in Tab. AN2-1. Amplicons were purified with the High Pure clean-up kit (Roche) and cloned in pGem-T Easy vectors (Promega). Plasmids were transformed into DH10B cells, singularised, purified with the GeneJET Plasmid Miniprep Kit (Thermo Fischer) and Sanger-sequenced to verify the correct inserts (StarSEQ).

Tab. AN2-1: Primer sequences used for RT-PCR and quantitative RT-PCR for *Spalax galili* (Sga) and *Rattus norvegicus* (Rno)

Primer	Sequence 5' > 3'	Amplicon size (bp)
A2M Sga for	CTTAATATCAGTTACACAGGGAG	150
A2M Sga rev	ATGGTTGTTGCTGACTTCAGTC	150
ATR Sga for	TAAGGAAAAGGGAATGTATATGAC	152
ATR Sga rev	GGAATGTTCTTAGAAACCACTCA	152
CISD2 Sga for	GCTCGCCTCACAGTTTCAG	161
CISD2 Sga rev	TTCACCACTTTGGGATTTTCCTT	161
WRN Sga for	CCCATCAACTCAGATATGTATAAA	150
WRN Sga rev	TCTCTTCCTGTTGGAACCACA	150
FGF21 Sga for	CCTGGAAATTAGGGCAGATG	171
FGF21 Sga rev	TCAAAGTGAGGCGATCCATAC	171
XPA Sga for	CCACTGGAGGCATGACCAA	163
XPA Sga rev	CCACACTCTTCACATATCAAGT	163
TOP2B Sga for	TGAATCTAACATCATCAGCATTTG	133

TOP2B Sga rev	GTTGCGGCCACCTGTAAC	133
CITED2 Sga for	ATCGCAGCCTCGAGCGCT	130
CITED2 Sga rev	CCATTTCCAGTCCTTCCGTC	130
GNMT Sga for	CTACAAGAGTGACCTGACCA	155
GNMT Sga rev	GTGGGTAGTAAGAGAGCCG	155
PTEN Sga for	CGGAACTTGCAATCCTCAGT	147
PTEN Sga rev	AACTCTACTTTGATGTCACCAC	147
VEGFA Sga for	TCTGGGTATGGCTGGCTG	142
VEGFA Sga rev	TTCTTTGGTCTGCATTCACATC	142
A2M Rno for	CCACCCAGGACACTGTAGT	146
A2M Rno rev	TAATTGGTTGTTGTTGTTGACTTG	146
ATR Rno for	GTCAATGAGAAGGCTAAGACC	169
ATR Rno rev	AACCAAGGTACATCTGACATAG	169
CISD2 Rno for	GAAGAAGAAGCAACAGAAGGAT	143
CISD2 Rno rev	CAGGGAACGTCTTGGAGC	143
WRN Rno for	TCCCATCAACTCAGATATGTATAA	158
WRN Rno rev	GGAGACACCTCTTCCTGTTG	158
FGF21 Rno for	AGATCAGGGAGGACGGAAC	163
FGF21 Rno rev	AAGTGAGGCGATCCATAGAGA	163
XPA Rno for	CGGAGGCGTGACCAGCAT	166
XPA Rno rev	CTCTTTCCCACACTCTTCACA	166
TOP2B Rno for	GACCTGGGTGAACAATGCTG	172
TOP2B Rno rev	CATTAACTGTGTCAGTGGTTCC	172
CITED2 Rno for	TGAGGAGCGGCTAGGGCA	213
CITED2 Rno rev	CATTTCCAGTCCTTCCGTCT	213
GNMT Rno for	CACCCCCAGGGAAGAACA	160
GNMT Rno rev	CGAAACTTACTGAAGCCAGG	160
PTEN Rno for	CTTGCAATCCCCAGTTTGTG	149
PTEN Rno rev	GTGGAAGAACTCTACTTTGATG	149
VEGFA Rno for	GATGAAGCCCTGGAGTGC	135
VEGFA Rno rev	CTTTGGTCTGCATTCACATCTG	135

Spalax and rat cDNAs were quantified by qRT-PCR with the GoTaq qPCR Master Mix (Promega) at a total volume of 10 µl and at an annealing temperature of 58°C in an ABI 7500 Fast Real Time PCR system (Applied Biosystems). Primers were used as listed in Tab. AN2-1. Amplicons were measured in triplicates and quantified by calibration on according standard curves which were measured in serial 10-fold dilutions. Relative expression values were calculated with Excel 2013 (Microsoft) and compared to relative FPKM values from the RNA-Seq analysis. As internal controls for checking RT efficiencies, RNA samples were spiked with 60ng of *Drosophila melanogaster (Dme)* RNA. During qRT-PCRs, we additionally ran an assay on the *Dme* Glob1 gene to approve that equivalent amounts of cDNA were synthesised for all samples.

For all *Spalax* and rat liver samples, we chose a subset of 11 genes to evaluate relative differential expression by qRT-PCR as previously predicted by RNA-Seq analyses. The tested genes covered represented the categories “cancer”, “ageing”, “DNA repair” and “hypoxia”. In the vast majority of experiments (23 out of 25), the direction (ratio) of differential expression between *Spalax* and rat or between normoxic and hypoxic samples of the same species agreed very well with the expression changes observed by RNA-Seq (Tab. AN2-2). In particular, elevated normoxic transcript levels in *Spalax* versus rat were displayed as expected by six genes, highly important for the inter-species comparison (*A2M*, *ATR*, *CISD2*, *WRN*, *XPA*, *TOP2B*). Unfortunately, due to shortage in RNA/cDNA availability for *Spalax* samples, not all of the 11 genes could be compared across the two species. Analysing the hypoxia response, the inducibility of *FGF21* and *A2M* in *Spalax*, and of *CITED2*, *ATR*, *VEGFA* and especially *A2M* in rat was also confirmed by qRT-PCR (Fig. AN2-1). Significance of gene expression differences was tested applying two-sided t-tests in Excel (Microsoft).

Tab. AN2-2: Relative expression of selected candidate genes between hypoxic (Hx) and normoxic (Nx) *Spalax galili* and *Rattus norvegicus* liver samples quantified by qRT-PCR and RNA-Seq. Green shading indicates matching results, grey shading indicates diverging results, *= high ratio due to division by virtually no FPKM at normoxia, **= overall low expression in RNA-Seq, ***= *Spalax* RNA RIN values were lower indicating high degradation

**gene**	**category**	*Spalax* Hx/Nx		Rat Hx/Nx		*Spalax* Nx/Rat Nx	
		qRT-PCR	RNA-Seq	qRT-PCR	RNA-Seq	qRT-PCR	RNA-Seq
A2M	cancer/ageing	1.4	1.6	94.1	3,056.4*	1517.7	63,475.0
ATR	repair/ageing	1.2	1.0	1.5	1.5	28.6	20.5
CISD2	ageing	1.3	0.9	1.7	1.5	38.9	12.3
FGF21	ageing	289.4	123.0	1.7	0.8	1.1**	0.1**
WRN	repair/ageing/ cancer	1.2	1.1	0.5	0.9	39.3	14.9
XPA	repair/ageing	0.9	0.7	0.6	0.5	24.8	59.5
TOP2B	repair/cancer	1.1	0.7	0.8	1.7	121.8	38.9
CITED2	hypoxia	n.a.***	1	46.8	40.6	n.a.***	1.5
GNMT	cancer	n.a.***	1.2	2.1	1.3	n.a.***	16.1
PTEN	cancer	n.a.***	4.3	1.5	0.9	n.a.***	1.7
VEGFA	hypoxia	n.a.***	1.5	5.5	1.8	n.a.***	1.5

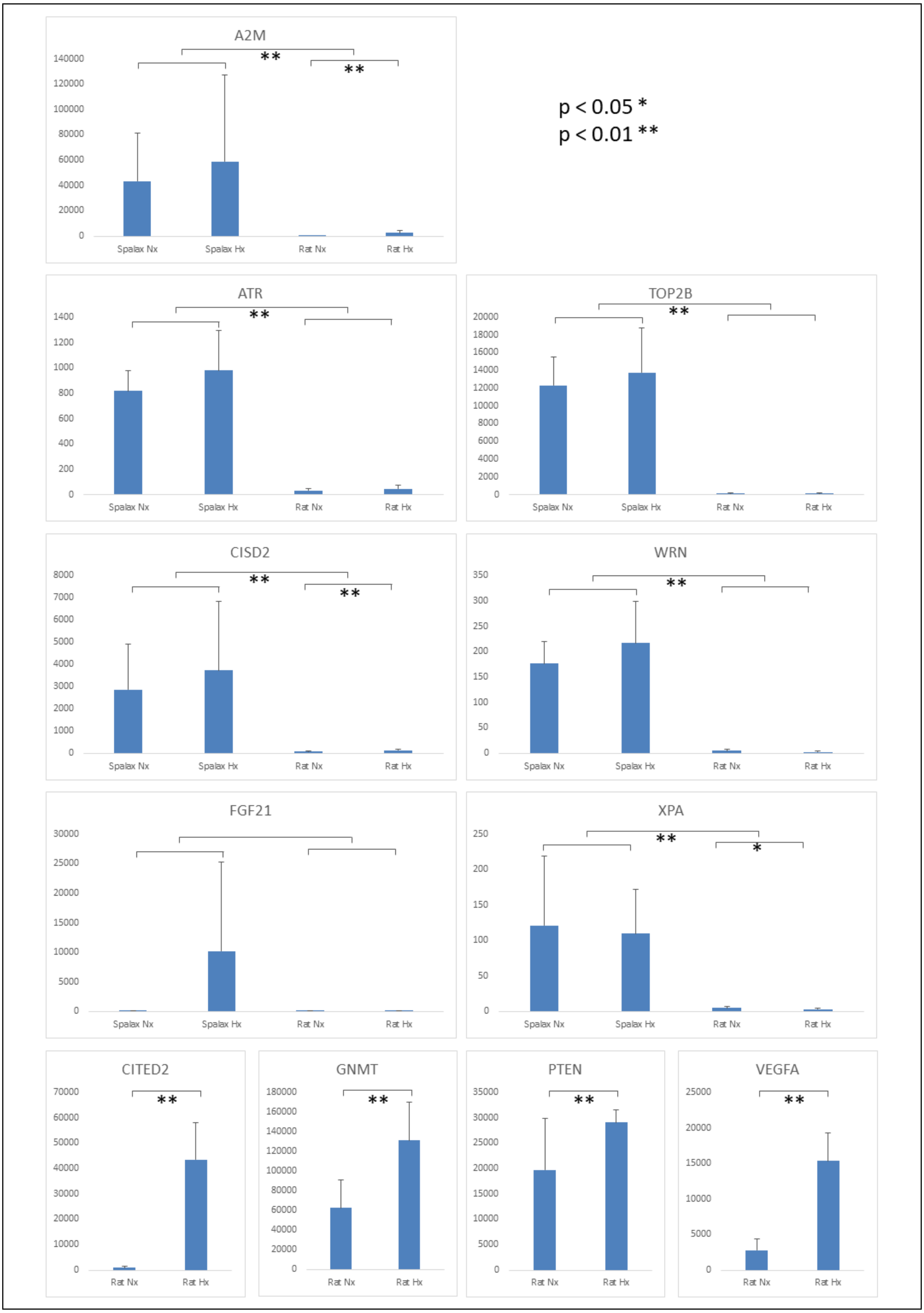

Fig. AN2-1: Results of the q-RT-PCR experiments.

References

1 Bustin, S. A. & Nolan, T. Analysis of mRNA expression by real-time PCR. *Real-time PCR: advanced technologies and applications. Caister Academic Press, Norfolk, United Kingdom*, 51-88, (2009).

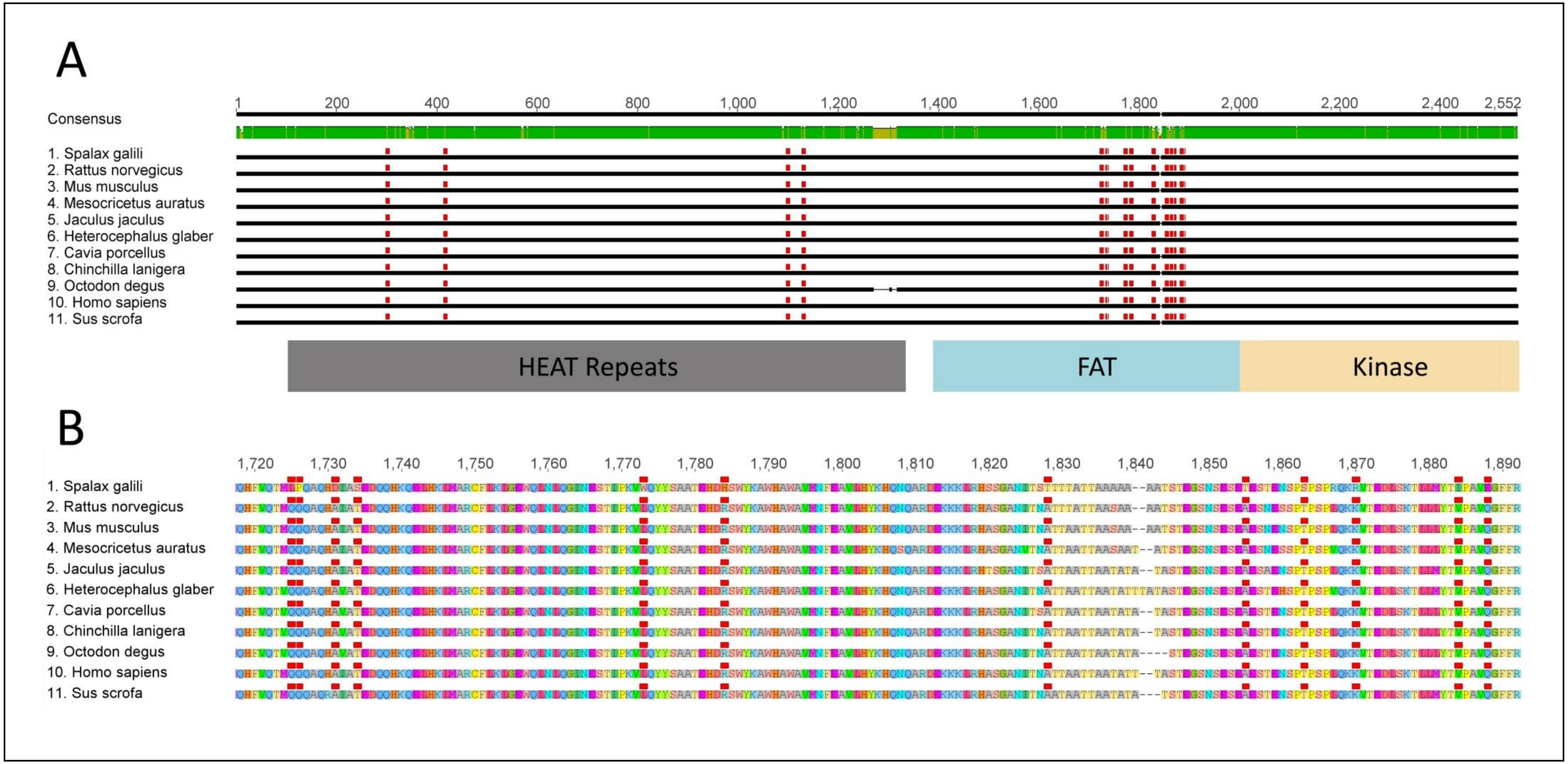

Additional Figure 2 – Amino acid replacements in mTOR

In mTOR 17 *Spalax*-specific amino acid replacements were detected (positions where all other mammals under study have a fixed amino acid). A: Domain structure of mTOR. 12 of the amino acid replacements are located within the FAT domain of the enzyme. B: Amino acid alignment; detail of the replacement-rich area of the FAT domain. The 17 replacements: K301R, T416A, I1100V, S1131L, K1133R, Q1725L, Q1726P, A1731D, T1734S, L1773W, R1784H, A1828T, A1855T, T1863S, K1870R, V1884I, Q1888E.
